# Analysis of *CFB*, a cytokinin-responsive gene of *Arabidopsis thaliana* encoding a novel F-box protein regulating sterol biosynthesis

**DOI:** 10.1093/jxb/erx146

**Published:** 2017-05-12

**Authors:** Wolfram G Brenner, Jan Erik Leuendorf, Anne Cortleven, Laetitia B B Martin, Hubert Schaller, Thomas Schmülling

**Affiliations:** 1 Institute of Biology/Applied Genetics, Dahlem Centre of Plant Sciences (DCPS), Freie Universität Berlin, Albrecht-Thaer-Weg, Berlin, Germany; 2 Institut de Biologie Moléculaire des Plantes du CNRS, Université de Strasbourg, rue du Général Zimmer, Strasbourg Cedex, France

**Keywords:** Albinism, chloroplast, cycloartenol synthase, cytokinin, F-box protein, plastid, sterol biosynthesis

## Abstract

Protein degradation by the ubiquitin–26S proteasome pathway is important for the regulation of cellular processes, but the function of most F-box proteins relevant to substrate recognition is unknown. We describe the analysis of the gene *Cytokinin-induced F-box encoding* (*CFB*, AT3G44326), identified in a meta-analysis of cytokinin-related transcriptome studies as one of the most robust cytokinin response genes. F-box domain-dependent interaction with the E3 ubiquitin ligase complex component ASK1 classifies CFB as a functional F-box protein. Apart from F-box and transmembrane domains, CFB contains no known functional domains. *CFB* is expressed in all plant tissues, predominantly in root tissue. A *ProCFB:GFP-GUS* fusion gene showed strongest expression in the lateral root cap and during lateral root formation. CFB-GFP fusion proteins were mainly localized in the nucleus and the cytosol but also at the plasma membrane. *cfb* mutants had no discernible phenotype, but *CFB* overexpressing plants showed several defects, such as a white upper inflorescence stem, similar to the hypomorphic cycloartenol synthase mutant *cas1-1*. Both *CFB* overexpressing plants and *cas1-1* mutants accumulated the CAS1 substrate 2,3-oxidosqualene in the white stem tissue, the latter even more after cytokinin treatment, indicating impairment of CAS1 function. This suggests that CFB may link cytokinin and the sterol biosynthesis pathway.

## Teaser

Albinotic inflorescence stems of Arabidopsis plants overexpressing the cytokinin-responsive gene *Cytokinin-induced F-box encoding* (*CFB*) phenocopy the sterol biosynthesis mutant *cas1-1* and accumulate 2,3-oxidosqualene, suggesting a link between cytokinin and sterol biosynthesis.

## Introduction

An important mechanism by which organisms adjust cellular functions is the controlled, specific protein degradation by the ubiquitin–26S proteasome pathway (Ciechanover *et al.*, 2000; Pickart, 2001; Smalle and Vierstra, 2004). The critical step—the recognition and polyubiquitination of target proteins—is mediated by different types of E3 ubiquitin ligases. As constituents of the SCF-type E3 ligases, F-box proteins are responsible for substrate recognition, thereby enabling polyubiquitination of the target protein. By means of the F-box, they bind to the SKP (in Arabidopsis: ASK) protein of the E3 ligase complex (Bai *et al.*, 1996) and possess additional domains to interact with the substrate protein. The target proteins of most of the >700 F-box proteins of Arabidopsis are not known.

The plant hormone cytokinin exerts its functions mainly through transcriptional activation of its primary target genes, which are activated by type-B response regulators (Sakai *et al.*, 2000; Hwang and Sheen, 2001; Sakai *et al.*, 2001). These are activated by phosphorylation after the cytokinin signal has been transduced from sensor histidine kinase receptors to the nucleus by a multi-step His-Asp phosphorelay signaling system (Werner and Schmülling, 2009; Kieber and Schaller, 2014). This pathway has been extensively studied and is now well characterized. In contrast, signaling downstream of this initial pathway is only partially known. Transcriptomic approaches have shed light on cytokinin-regulated genes (Rashotte *et al.*, 2003; Brenner *et al.*, 2005, 2012; Bhargava *et al.*, 2013; Brenner and Schmülling, 2015). Besides some immediate early cytokinin response genes providing feedback to the upstream cytokinin metabolic and signaling system (type-A response regulator genes), most of them may contribute to physiological and developmental downstream responses of cytokinin (Argueso *et al.*, 2009; Werner and Schmülling, 2009; Ha *et al.*, 2012; Hwang *et al.*, 2012; Vanstraelen and Benková, 2012; El-Showk *et al.*, 2013; Kieber and Schaller, 2014). These cytokinin-regulated genes probably play a specific role in the execution of the multiple functions of cytokinin and are therefore primary candidates for further investigation. One of these cytokinin responsive genes is *CFB* (*Cytokinin-induced F-box encoding*), which was found in a meta-analysis of cytokinin-related transcriptome data (Brenner and Schmülling, 2015) and encodes a putative F-box protein.

In various hormonal pathways, polyubiquitination of target proteins by SCF-type E3 ligases mediated by specific F-box proteins plays an important role, for example, TIR1 (Gray *et al.*, 2001; Dharmasiri *et al.*, 2005; Kepinski and Leyser, 2005) and COI1 (Dai *et al.*, 2002; Xu *et al.*, 2002), regulating the auxin and jasmonic acid pathways, respectively. Few reports regarding the involvement of targeted protein degradation by the ubiquitin–proteasome pathway and its functional relevance for cytokinin signaling have been published, and those that exist have partially contradictory results (Smalle *et al.*, 1997; Yamada *et al.*, 2004; Kim *et al.*, 2013).

Here, we present the characterization of the above-mentioned cytokinin-regulated gene, *CFB.* Overexpression of *CFB* caused a pleiotropic phenotype with the development of albinotic tissue at the apical end of the inflorescence stem. The morphological, cytological, and chemical phenotypes of plants with enhanced *CFB* expression resembled those of the cycloartenol synthase mutant *cas1-1* (Babiychuk *et al.*, 2008*a*, 2008*b*). The phenotype and cytokinin-dependent hyperaccumulation of the CAS1 substrate 2,3-oxidosqualene in *cas1-1* mutants suggests a link between cytokinin signaling and sterol biosynthesis.

## Materials and methods

### Phylogenetic analysis and analysis of protein structure

Molecular phylogenetic analyses by the Maximum Likelihood method were carried out using MEGA version 5.05 (http://www.megasoftware.net/) (Tamura *et al.*, 2011). The evolutionary history was inferred using the Maximum Likelihood method based on the JTT matrix-based model (Jones *et al.*, 1992). The bootstrap consensus tree inferred from 500 replicates (Felsenstein, 1985) is taken to represent the evolutionary history of the taxa analyzed (Felsenstein, 1985). Branches corresponding to partitions reproduced in less than 50% bootstrap replicates are collapsed. The percentage of replicate trees in which the associated taxa clustered together in the bootstrap test (500 replicates) are shown next to the branches (Felsenstein, 1985). Initial tree(s) for the heuristic search were obtained automatically by applying Neighbor-Join and BioNJ algorithms to a matrix of pairwise distances estimated using a JTT model and then selecting the topology with superior log likelihood value. The analysis involved 53 amino acid sequences. All positions containing gaps and missing data were eliminated. There was a total of 19 positions in the final dataset.

Multiple sequence alignments were carried out using MultAlin (http://multalin.toulouse.inra.fr/multalin/) with the standard settings (Corpet, 1988).

Domains were searched using NCBI CDD (Marchler-Bauer *et al.*, 2015), EMBL-EBI Pfam (Finn *et al.*, 2014), ExPASy Prosite (Sigrist *et al.*, 2013), and KEGG Motif search (Ye *et al.*, 2012). Alignments were carried out with MUSCLE at EBI (Edgar, 2004; Li *et al.*, 2015).

### Gene cloning and transformation procedures

DNA cloning procedures using the GATEWAY® system (Hartley *et al.*, 2000) are summarized in Supplementary Table S1 at *JXB* online. *Arabidopsis thaliana* was transformed by *Agrobacterium tumefaciens*-mediated transformation using the floral dip method (Clough and Bent, 1998).

### *cfb* mutant lines

The *cfb-1* (SAIL_215_B11) and *cfb-2* (SALK_205373) mutant lines were obtained from the Nottingham Arabidopsis Stock Centre (NASC, Nottingham, UK). The point of insertion was determined by sequencing, using the appropriate T-DNA-specific primers listed in Supplementary Table S2. Real-time quantitative RT-PCR (qRT-PCR) was carried out with 40 cycles of amplification (*T*_a_=55 °C, *t*_A_=1:15 min).

### Real-time quantitative RT-PCR

RNA was extracted as described previously (Brenner *et al.*, 2005). cDNA was synthesized using the SuperScript^®^ III reverse transcriptase (Invitrogen, Carlsbad, CA, USA), primed with a mixture of oligo-dT and N_9_ primers. qRT-PCR was carried out as described previously (Brenner and Schmülling, 2012). *UBC10* (AT5G53300) and *PP2AA2* (AT3G25800) were used as reference genes. Primers were designed using NCBI Primer-BLAST (Ye *et al.*, 2012) and are listed in Supplementary Table S2.

### Histochemical staining procedures and microscopy

Histochemical analysis of the β-glucuronidase (GUS) reporter enzyme was performed according to (Jefferson *et al.*, 1987; Werner *et al.*, 2003) for the times indicated in Fig. 3 and Supplementary Fig. S1.

3,3ʹ-Diaminobenzidine (DAB) staining was performed according to Daudi and O’Brien (2012), modifying the infiltration procedure (vacuum application three times until boiling of the solution) and the incubation time (overnight) to account for the more rigid stem and pedicel tissue.

To detect lignification, 1 g phloroglucinol was dissolved in a mixture of 40 ml 20% ethanol and 10 ml 32% hydrochloric acid. The tissue was stained directly under the microscope.

The samples were inspected with a Zeiss Axioskop 2 plus microscope with Plan-Apochromat 20× and Plan-Neofluar 40× objectives. Images were obtained with an AxioCam ICc3 camera, and captured and post-processed with AxioVision software version 4.6.1.0 (Carl Zeiss Microscopy GmbH, Jena, Germany). The stereo microscope used was an Olympus SZX12 with a UC30 camera.

### Quantitative β-glucuronidase assay

Assays were conducted according to (Jefferson *et al.*, 1987; Gallagher, 1992) adapted to volumes compatible with 96-well microtiter plates. Protein concentrations were determined by the Bradford assay (Jones *et al.*, 1992). GUS activity was quantified as MUG-MU conversion per mg protein per min and normalized to the untreated control. Statistical significance of differences was tested with Student’s *t*-test.

### Transient leaf transformation and confocal fluorescence microscopy

*Nicotiana benthamiana* leaves were infiltrated with the *Agrobacterium tumefaciens* strain GV3101 carrying *CFB-GFP* constructs (Supplementary Table S1) using a published protocol (Sparkes *et al.*, 2006).

Plasma membranes were stained by infiltrating the dye FM4-64 (50 µM, Invitrogen, Carlsbad, CA, USA) into *N. benthamiana* leaf epidermal cells. After incubation for 10 min, samples were inspected using a Leica TCS SP5 confocal unit attached to a Leica DMI6000 CS microscope. The 488 nm laser line was used for excitation. Emission was detected between 500 and 530 nm or 625 and 665 nm, respectively (Wulfetange *et al.*, 2011). Images were acquired using LAS AF software version 2.7.3.9723 (Leica Microsystems GmbH, Wetzlar, Germany).

### Transmission electron microscopy

Part of the stems of *Pro35S:CFB* and wild-type plants were fixed for 3 days at 4 °C using vacuum infiltration in 2% (v/v) paraformaldehyde, 2% (v/v) glutaraldehyde buffered in 50 mM cacodylate buffer with 50 mM NaCl. Samples were washed with 50 mM cacodylate buffer containing 50 mM NaCl and with 50 mM glycylglycine buffer containing 100 mM NaCl. Postfixation was performed in 1% (w/v) osmium tetroxide buffered in 50 mM cacodylate buffer containing 50 mM NaCl for 3 h. After washing with distilled water, leaf tissues were incubated for 1 h in 0.1% (w/v) tannic acid in 100 mM HEPES buffer, rinsed with water, and incubated overnight at 4 °C in water. After staining in 2% (w/v) uranylacetate for 1.5 h, fixed tissues were dehydrated and embedded in Spurr’s epoxy resin. Ultra-thin sections (65 nm), obtained using a Leica Ultracut UCT ultramicrotome, were mounted on 0.7% (w/v) formvar coated copper grids, 200 mesh. The sections were contrasted with uranyl acetate [2% (w/v) in 50% ethanol] followed by lead citrate [4% (w/v) solution] and examined in a FEI Tecnai Spirit transmission electron microscope operated at 120 kV.

### Protein purification and protein blot analysis

Plant proteins of *in vitro* grown 10-d-old *Arabidopsis* seedlings were extracted by using a microsomal purification protocol (Kasaras and Kunze, 2017). Plant material (0.5 g) was ground in liquid nitrogen using a mortar and pestle and resuspended in 3–5 ml ice-cold extraction buffer [50 mM HEPES pH 6.5, 5 mM EDTA pH 8.0, 10% sucrose, 1 mM DTT, protease inhibitor cocktail (complete mix, Roche Applied Science, Penzberg, Germany) and 1 mM PMSF]. The solution was filtered through a double layer of miracloth and spun down by ultracentrifugation (100000 *g* for 1 h at 4 °C). The supernatant was decanted and analyzed separately. The pellet was suspended in 50 µl extraction buffer by pipetting and subsequently mixed with 2× Laemmli sample buffer (Laemmli, 1970). Supernatant and pellet fractions were separated in an SDS-polyacrylamide gel and blotted on to a polyvinylidene difluoride (PVDF) membrane. GFP fusion proteins were detected using the anti-GFP antibody [3H9] (Chromotek, Planegg-Martinsried, Germany).

Yeast proteins were extracted as described by Kushnirov (2000) and also separated in an SDS-polyacrylamide gel and blotted on to a PVDF membrane. LexA-DB:CFB and Gal4-AD:ASK1 fusion proteins were detected using LexA (sc-7544) and Gal4-AD antibodies (sc-1663), respectively (Santa Cruz Biotechnology Inc., Dallas, TX, USA).

Detection and visualization were performed with a chemiluminescence kit (SuperSignal™ West Pico Chemiluminescent Substrate, ThermoFisher Scientific, Waltham, MA, USA) and standard autoradiography film. After immunodetection, the membrane was stained by Coomassie stain (stain: 25% isopropanol, 10% acetic acid, and 0.05% Coomassie-R-250; destain: 50% ethanol, 10% acetic acid) as a control for equal protein loading.

### *In vivo* protein interaction studies

For yeast two-hybrid analyses, a *lexA*-based system was used as described previously (Leuendorf *et al.*, 2008). The cDNAs of the *ASK1* (AT1G75950) and *CFB* (AT3G44326) genes were cloned into *pDONR221* (Invitrogen) and introduced into the plasmids *pBTM116-D9* and *pACT2* (Clontech, Mountain View, CA, USA) (GenBank accession no. U29899), respectively, modified to be compatible with the GATEWAY system (Invitrogen, Carlsbad, CA, USA). Vectors were transformed into yeast L40ccU3 cells (Goehler *et al.*, 2004) as previously described (Gietz and Woods, 2002). Cells were grown on SD minimal agar (Sambrook and Russell, 2001) with Leu and His (SDII). Colonies were diluted 1:100 to 1:10000 in autoclaved distilled water before transfer to SD minimal media without supplements (SDIV) for testing protein interaction. Photographs were taken after 3 d of incubation at 28 °C.

For the split-ubiquitin-based analyses (Snider *et al.*, 2010), CFB was fused to the C-terminal part of ubiquitin (Cub) by cloning the cDNA without the stop codon into the vector pMetYC_GW (TAIR strain CD3-1740) (Obrdlik *et al.*, 2004). ASK1 was fused to the non-interacting N-terminal mutant part of ubiquitin (NubG) by introducing the cDNA into the vector pNX32_GW (TAIR strain CD3-1737) (Obrdlik *et al.*, 2004). For positive and negative controls, CFB-Cub was tested for interaction either with the interacting N-terminal part of ubiquitin (NubI) by using the empty vector pNWT-X_GW (TAIR strain CD3-1739) (Obrdlik *et al.*, 2004), or with NubG by using the empty vector pNX32_GW. The yeast reporter strain THY.AP4α (Obrdlik *et al.*, 2004) was transformed as described above. Yeast cells were grown on SD media with complete supplement mixture (CSM) drop-out –Ade, –His, –Leu, –Met, –Trp, –Ura (Formedium, UK), 0.002% adenine, and 0.002% histidine (SD –L, –W). Interaction was screened on SD media containing only CSM drop-out and 135 µM Met (SD –L, –W, –A, –H, 135 µM Met).

### Cytokinin induction and measurement of sterol metabolites

Adult plants for induction were grown on soil in a greenhouse until roughly 50% of the flowers were open. The plants were then sprayed with a solution of 5 µM 6-benzyladenine containing 0.01% DMSO as solvent and carrier three times per day (in the morning, at noon, and in the evening) for 3 days. On the fourth day of treatment, the plants were sprayed one more time, 2 h before the upper third of the inflorescence stems, which is the white part in *cas1-1* mutants, was harvested. The samples were collected in three replicates, each containing material from at least four individual plants, frozen in liquid nitrogen, stored at –80 °C, and freeze-dried before extraction. Samples of 13–150 mg (dry weight) of tissues were extracted according to Babiychuk *et al.* (2008*a*) with some modifications. Briefly, the samples were saponified in 15 ml 6% KOH in MeOH at 70 °C for 2 h. The nonsaponifiable compounds were extracted twice with 20 ml *n*-hexane and, after evaporation of the *n*-hexane, resuspended in dichloromethane, and dried again. After derivatization (1 h at 70 °C in 100 µl toluene, 50 µl acetic anhydride, and 30 µl pyridine), the organic extracts were analyzed by GC-MS [Agilent 6890 gas chromatograph and 5973 mass selective detector equipped with a HP5-MS column (J&W; 30 m long, 0.32 mm internal diameter, 0.25 µm film thickness)] and quantified by GC-FID [Agilent 6890 gas chromatograph equipped with a flame-ionization detector and a DB5 column (J&W; 30 m long; 0.32 mm internal diameter, 0.25 µm film thickness)]. Gas chromatography parameters were as described in Babiychuk *et al.* (2008*a*).

## Results

### Discovery of the cytokinin-regulated *CFB* gene

**Fig. 1.**
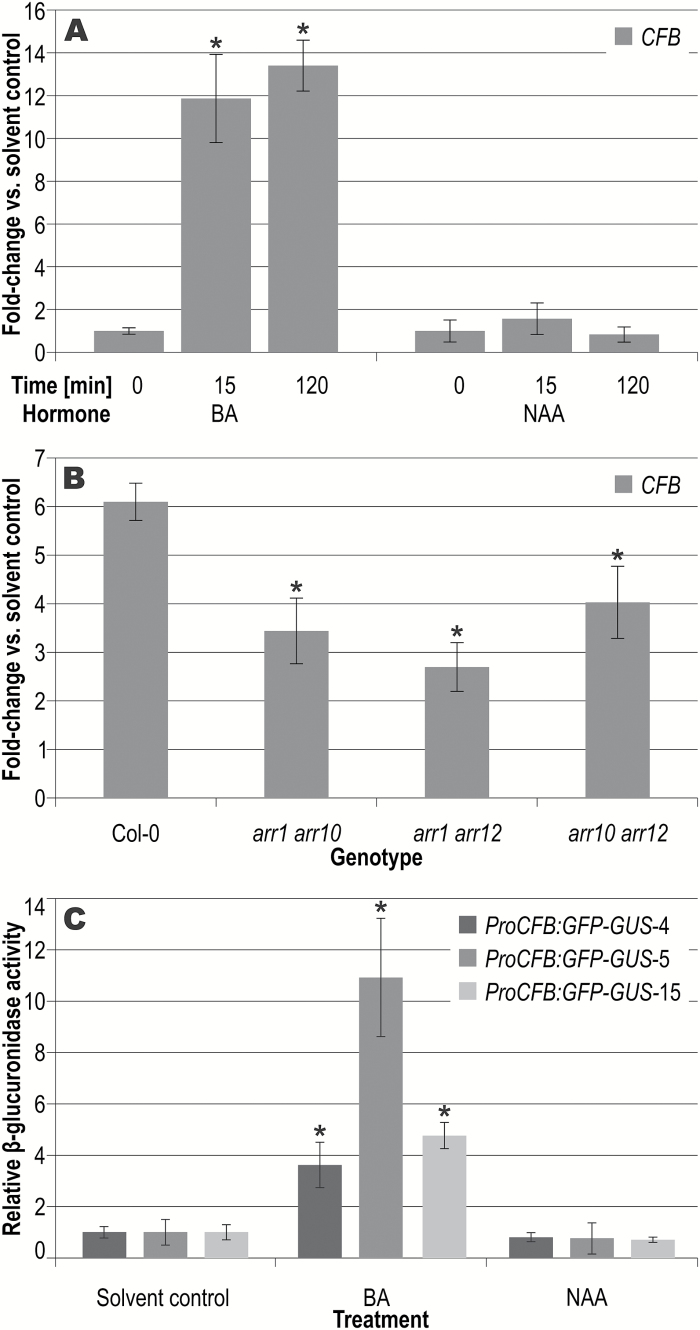
Cytokinin responsiveness of the *CFB* gene. (A) Transcript levels of *CFB* were quantified by qRT-PCR in 7-d-old Col-0 seedlings after 15 min or 2 h of treatment with cytokinin (5 µM 6-benzyladenine; BA) or auxin (5 µM 1-naphthaleneacetic acid; NAA), or 2 h with the solvent (time=0 min). Error bars=SD (n=3). (B) Transcript levels of *CFB* in seedlings of three type-B response regulator (*ARR*) double mutant lines and Col-0 were quantified by qRT-PCR after 2 h of treatment with cytokinin or the solvent. Error bars=SD (n=3). (C) 11-d-old Arabidopsis seedlings of three independent lines carrying a *ProCFB:GFP-GUS* fusion gene were treated for 6 h with either 1 µM BA or 1 µM NAA. Relative GUS activity of three independent lines was analyzed by a quantitative MUG assay in comparison to the untreated control (solvent control), which was set to a value of 1. Error bars=SD (n=6). Asterisks indicate significant differences relative to the solvent control or to the wild type, respectively (Student’s *t*-test; *P*<0.001 for A and C, *P*<0.05 for B).

The gene *AT3G44326* was found to be a cytokinin-regulated gene in a meta-analysis of CATMA (Crowe *et al.*, 2003; Allemeersch *et al.*, 2005) microarray data, ranking second after the type-A response regulator gene *ARR6* (Brenner and Schmülling, 2015). Its earlier identification as a cytokinin-regulated gene was prevented by its absence on the Affymetrix ATH1 array used for most cytokinin-related microarray studies and previously published meta-analyses (Brenner *et al.*, 2012; Bhargava *et al.*, 2013). The cytokinin responsiveness of the *AT3G44326* transcript level was verified in Arabidopsis seedlings using both qRT-PCR and transgenic plants harboring a reporter gene consisting of a ~2 kb genomic fragment upstream of the *CFB* gene and a *GFP-GUS* fusion gene (*ProCFB:GFP-GUS*) (Fig. 1). Shortly (15 min) after cytokinin treatment, the mRNA level of *AT3G44326* was increased ~14-fold, characterizing *CFB* as an immediate-early cytokinin response gene. The rapid induction of *AT3G44326* by cytokinin was also confirmed by RNA sequencing (RNA-seq), where the abundance of the corresponding transcript was found to be increased 13.4-fold by cytokinin (Bhargava *et al.*, 2013). The expression level was further increased after 2 h of cytokinin induction (Fig. 1A). The induction of *CFB* by cytokinin was attenuated in all three double mutants of the *ARR1*, *ARR10*, and *ARR12* genes, which encode type-B response regulators, the class of transcription factors mediating the major part of the transcriptional response to cytokinin during vegetative growth. This corroborates the idea that the *CFB* gene is directly regulated by the phosphorelay cytokinin signaling system (Fig. 1B). In accordance with the qRT-PCR results, plants harboring the *ProCFB:GFP-GUS* reporter gene showed a significantly enhanced GUS activity following cytokinin treatment in a quantitative MUG assay (Fig. 1C) and in histochemical analyses (Supplementary Fig. S1). Here, GUS staining was more intense after cytokinin treatment and remained restricted to the root. In contrast, treatment with the synthetic auxin naphthaleneacetic acid neither had a significant effect on the transcript level of the gene nor showed an increase in GUS activity in *ProCFB:GFP-GUS* reporter lines, confirming the specificity of the response of the gene to cytokinin (Fig. 1A, C).

### CFB and two related proteins form a distinct group among the F-box proteins having no known protein–protein interaction domain

**Fig. 2.**
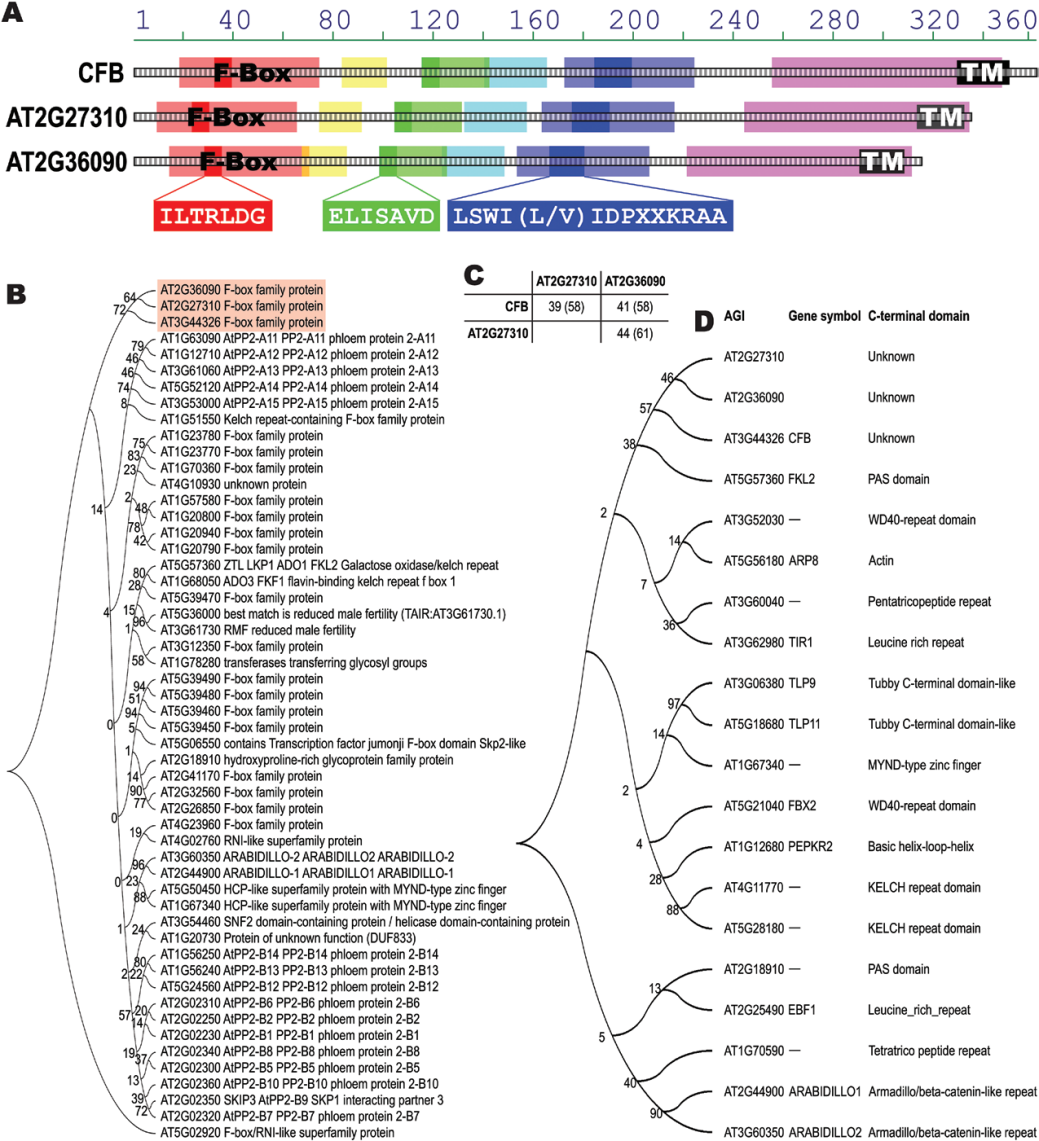
Sequence analysis of CFB, AT2G27310, and AT2G36090 proteins. (A) Structure of conserved regions in CFB, AT2G27310, and AT2G36090. Blocks of similar sequences were identified using the ClustalW implementation AlignX Blocks (InforMax Inc., Bethesda, MD, USA) and are marked in light red, yellow, green, cyan, blue, and magenta. The light red sequence block is identical to the annotated F-box domain. The conserved sequence motifs unique to the CFB subfamily of F-box proteins, ILTRLDG, ELISAVD, and LSWI(L/V)IDPXXKRAA, are highlighted in solid red, green, and blue, respectively. Predicted membrane-spanning regions are represented as black boxes (labeled TM). (B) Molecular phylogenetic analysis by the Maximum Likelihood method, using the whole protein sequences of CFB, AT2G27310, and AT2G36090 in relation to the members of family E of the F-box superfamily. Numbers at the branching points are bootstrap values. (C) Percentages of identical and similar (in brackets) amino acids shared by CFB, AT2G27310, and AT2G36090. (D) Molecular phylogenetic analysis by the Maximum Likelihood method using the protein sequences C-terminal to the F-box domains of CFB, AT2G27310, and AT2G36090 in relation to representative members of the F-box superfamily containing different C-terminal domains. Numbers at the branching points are bootstrap values. The trees in B and D were generated using MEGA version 5 (Tamura *et al.*, 2011).

DNA sequence analysis of the *CFB* gene predicts a single exon without any introns. The protein encoded by this gene has 363 amino acids and contains an F-box domain extending from amino acid 36 to 67 (Fig. 2A). Apart from a predicted α-helical transmembrane domain close to the C-terminal end, there are no known or predicted domains based on analyses using the Aramemnon database (Schwacke *et al.*, 2003) and the pertinent online search tools (see Materials and methods).

Sequence analysis showed that the proteins most closely related to CFB are encoded by *AT2G27310* and *AT2G36090*. All three proteins contain, in addition to the F-box, five conserved regions C-terminal of the F-box domain (Fig. 2A).

The phylogenetic relationships of the F-box superfamily of proteins in Arabidopsis have been investigated (Gagne *et al.*, 2002), but CFB was missing in the study because the encoding gene was not annotated at that time. According to this study, AT2G27310 and AT2G36090 belong to family E of the F-box proteins. To fit CFB into this protein family, we performed an alignment of all family E F-box proteins identified previously (Gagne *et al.*, 2002), including CFB (Fig. 2B), confirming the close relationship of CFB with AT2G27310 and AT2G36090. The three proteins appear to form a distinct subgroup within family E of the F-box proteins.

Sequence alignment of the three members of the CFB subgroup in Arabidopsis and their orthologs in other plant species identified three conserved motifs, which are not present in any other Arabidopsis F-box protein: ILTRLDG within the F-box domain, and ELISAVD and LSWI(L/V)IDPXXKRAA, both located C-terminal of the F-box domain (Fig. 2A, Supplementary Fig. S2).

Typically, F-box proteins contain one or more protein–protein interaction domains C-terminal to the F-box domain (Kipreos and Pagano, 2000; Gagne *et al.*, 2002), by which they are grouped into structural classes. To assign the three proteins of the CFB subgroup to one of these structural classes, another phylogenetic analysis was carried out with representative members of different structural classes using the protein sequences C-terminal to the F-box domain. This analysis corroborated the finding that the three proteins of the CFB family form a distinct structural class and do not belong to one of the known major structural classes of F-box proteins (Fig. 2D). It also confirmed that CFB does not contain one of the major known protein–protein interaction domains.

### The *CFB* gene is predominantly expressed in root tissue

**Fig. 3.**
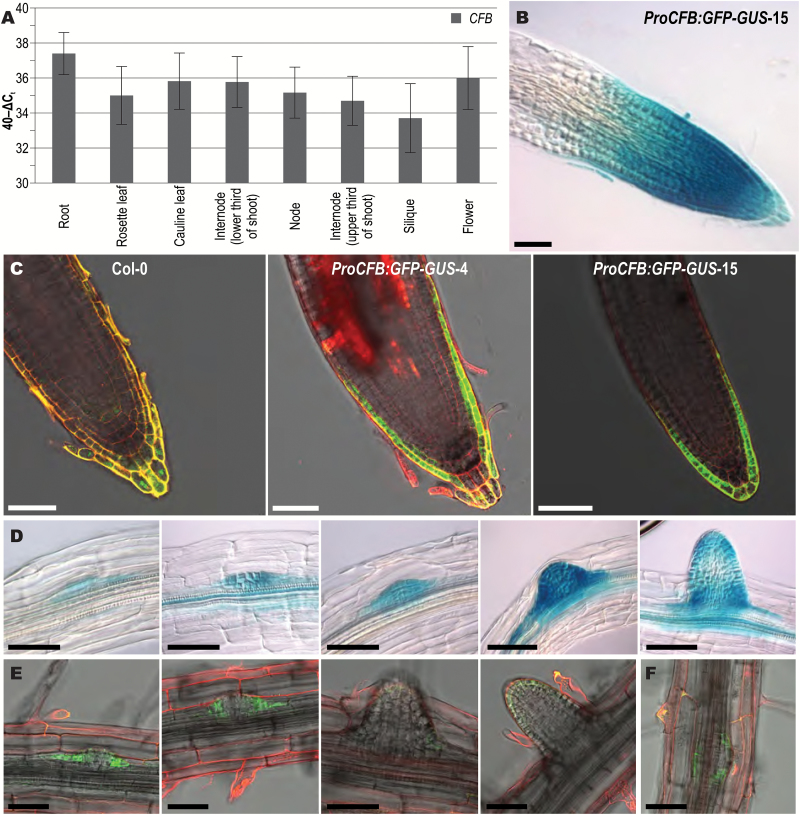
Expression pattern of the *CFB* gene. (A) Steady-state transcript levels of *CFB* in different plant tissues. The relative transcript levels were determined by qRT-PCR on total RNA. Error bars indicate SD (n=3). Internode (lower third) and Internode (upper third) refer to internodes in the lower or upper thirds of the stem, respectively. No significant differences were found (Student’s *t*-test, *P*<0.05). B–F, Expression pattern of a *ProCFB:GFP-GUS* reporter gene. (B) GUS staining of the root tip. (C) GFP fluorescence localized to the lateral root cap and the outer tier of the columella, in the primary root tips of wild type (Col-0) and two transgenic lines carrying a *ProCFB:GFP-GUS* gene (lines 4 and 15). (D) GUS staining of a series of lateral root primordia at different stages. (E) GFP fluorescence of the cells at the base of lateral root primordia. (F) GFP fluorescence of a ring of cells around the base of a lateral root primordium, viewed from the top. The root tissue shown in B and D was stained for 4 h. Bars=50 µm.

Because the major public databases are based on data yielded by the Affymetrix ATH1 array, which lacks probe sets for the *CFB* gene, no data are available on the developmental and tissue-specific pattern of *CFB* gene expression. qRT-PCR analysis of various plant organs showed that *CFB* mRNA was detectable in all organs assessed, with roots showing the highest expression level (Fig. 3A). Expression analysis using the *ProCFB:GFP-GUS* reporter gene showed a comparable result in three independent transgenic lines. GUS staining was strongest in the root tips but not detected in the shoot (Fig. 3B). Optical sections obtained by confocal fluorescence imaging revealed that the expression of the reporter gene in the root tip was mainly localized to the lateral root cap (Fig. 3C), partially overlapping with the expression pattern shown for the *TCS::GFP* cytokinin reporter (Zürcher *et al.*, 2013). In contrast to the *TCS::GFP* reporter, *ProCFB:GFP-GUS* expression was also visible in the lateral root primordia, starting concurrently with the first cell divisions and being present throughout the following developmental phases (Fig. 3D, E). The activity of the reporter gene appears to form a ring around the basis of the lateral root primordia and subsides as the lateral roots begin to emerge. Support for the root as the main expression site of *CFB* also comes from RNA-seq-based expression data (Cheng *et al.*, 2017) accessible at the Araport ThaleMine database (https://apps.araport.org/thalemine/).

### Subcellular localization of CFB-GFP fusion proteins

**Fig. 4.**
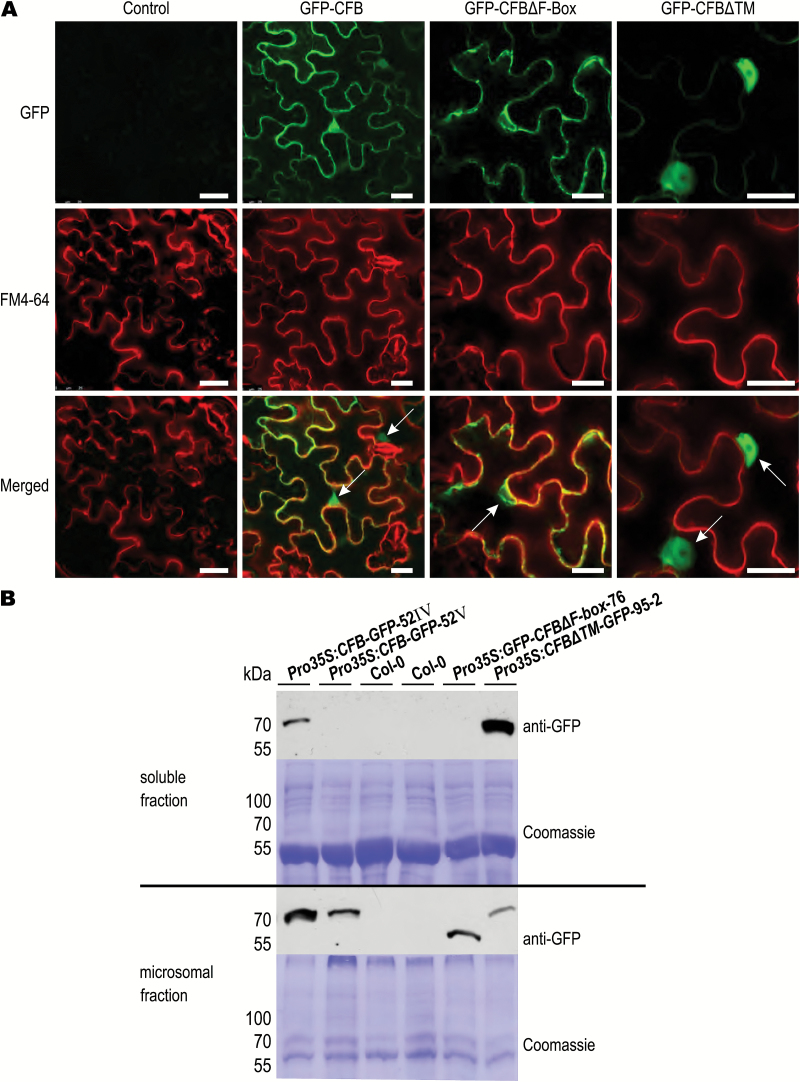
Subcellular localization of GFP-CFB fusion proteins. (A) The subcellular localization of N-terminal GFP fusion constructs using the full-length and truncated versions of CFB was examined in transiently transformed *N. benthamiana* leaves. Truncated versions lack the F-box (ΔF-box) or the predicted transmembrane domain (ΔTM), respectively. Fluorescence in the green channel represents the GFP signal; fluorescence in the red channel represents the plasma membrane marker FM4-64. Representative images are shown. Arrows point to the cell nuclei. Bars=25 µm. (B) Immunological detection of a GFP epitope in GFP-tagged CFB derivatives in the supernatant and the pellet after fractionation of protein extracts by ultracentrifugation and detection on protein blots. Contents of the lanes (left to right): two lanes with extracts of individual Arabidopsis plants expressing the GFP-tagged full-length CFB cDNA sequence, two lanes with wild-type (Col-0) extracts, one lane with an extract of a plant carrying a GFP-tagged CFB deletion construct lacking the F-box domain (ΔF-box), and one lane carrying a GFP-tagged CFB deletion construct lacking the C-terminal predicted transmembrane domain (ΔTM). Coomassie-stained membranes served as a loading control.

To determine the subcellular localization of CFB, we examined various GFP fusion constructs expressed transiently in *N. benthamiana* leaves by laser scanning microscopy. Fig. 4 shows that the subcellular localization of the fusion proteins appears to be determined by the N- and C-terminal regions of CFB. The signal of GFP-CFB fusion proteins containing the full-length CFB open reading frame appeared most strongly in the nucleus and at the plasma membrane, where it overlapped at least partially with the staining pattern of the membrane marker FM4-64 (Fischer-Parton *et al.*, 2000). GFP-CFB fusion proteins lacking the N-terminal 74 amino acids of CFB, including the F-box domain, were excluded from the nucleus, while the extranuclear signal distribution was not altered. Removal of the C-terminal 38 amino acids of CFB, containing the annotated transmembrane domain, caused the signal overlapping with the membrane marker FM4-64 to disappear. C-terminal GFP fusion constructs showed the same localization patterns (data not shown). The functionality of the GFP-fusion constructs containing the full-length CFB coding sequence was demonstrated in Arabidopsis plants stably overexpressing the fusion construct, which showed the same phenotype (see below) as plants overexpressing the native gene, albeit less severe (Supplementary Fig. S3).

We also analyzed the subcellular localization of the CFB-GFP fusion protein in stably transformed Arabidopsis seedlings. The overall expression of the CFB-GFP fusion protein was generally low and the GFP signal was difficult to detect, even after treatment with cytokinin or the proteasome inhibitor MG132. With the more sensitive immunodetection method we were able to detect the GFP epitope of CFB-GFP fusion proteins in microsomal fractions in which membrane-bound proteins are enriched (Fig. 4B). This is a clear indication that CFB is at least partially membrane localized. This was also true for the truncated version of CFB lacking the F-box domain (Fig. 4B). In contrast, the CFB protein lacking the transmembrane domain was enriched in the supernatant, indicating a localization distinct from the membrane (Fig. 4B).

### CFB interacts with ASK1, revealing it to be a structural constituent of an SCF-type E3 ubiquitin ligase

**Fig. 5.**
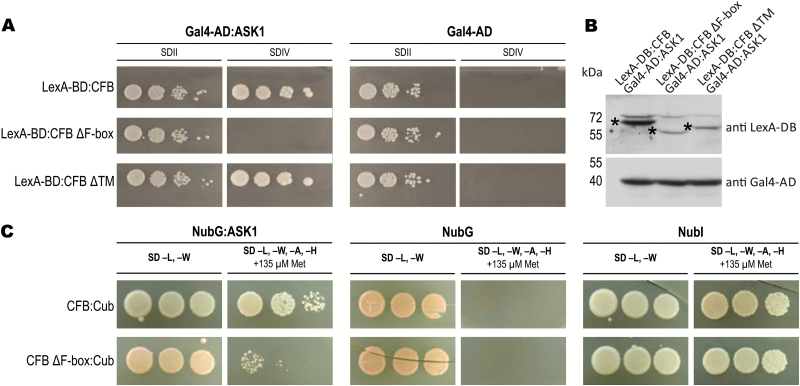
Interaction of CFB with the SCF E3 ubiquitin ligase complex component ASK1. (A) Interaction test using the yeast two-hybrid system. CFB and deletion versions, lacking the N-terminally located F-box (ΔF-box) or the C-terminal predicted transmembrane domain (ΔTM), fused to the LexA DNA-binding domain (LexA-BD), were tested for interaction against the ASK1 protein fused to the Gal4 activation domain (Gal4-AD) or, as a negative control, against Gal4-AD alone. Yeast cells were grown on control medium (SDII) and on selection medium for interaction studies without uracil and histidine supplements (SDIV), respectively. (B) Western blot to assess protein expression in the yeast strains used in A, confirming the expression and correct size of the tested yeast two-hybrid fusion proteins. Antibodies to LexA-DB and Gal4-AD were used for detection. Asterisks indicate the correctly sized LexA-DB:CFB fusion proteins. (C) Interaction test using the split-ubiquitin system. CFB and CFB ΔF-box fused to the C-terminal part of ubiquitin (Cub) were tested for interaction against a positive control consisting of the N-terminal interacting part of ubiquitin (NubI), a negative control consisting of the N-terminal non-interacting mutant part of ubiquitin (NubG), and ASK1 (NubG:ASK1). The interaction was tested on selection medium lacking leucine, tryptophan, adenine, and histidine (SD –L, –W, –A, –H), and supplemented with 135 µM methionine (+135 µM Met) to reduce the promoter activity of the CFB:Cub construct. The control medium was additionally supplemented with the amino acids uracil, histidine, and adenine (SD –L, –W). (This figure is available in colour at *JXB* online.)

Sequence analysis showed that CFB is a putative F-box protein. To obtain evidence for the functionality of CFB as a structural constituent of an SCF complex, we analyzed its interaction with the Arabidopsis SKP1 homolog ASK1 using yeast two-hybrid (Fig. 5A, B) and split-ubiquitin (Fig. 5C) assays. Both analyses showed that CFB binds in an F-box-dependent manner to ASK1, indicating that CFB is a functional F-box protein. Removal of the predicted transmembrane domain had no effect on the interaction between CFB and ASK1 (Fig. 5A). Notably, overexpression of N- and C-terminal deletion constructs lacking the F-box or the annotated transmembrane domain, respectively, never (i.e. none out of 150 or 85 T1 individuals, respectively) caused the phenotype induced by overexpression of the full-length CFB protein (see below). This corroborates the functional relevance of the F-box and the annotated transmembrane domains.

### T-DNA insertion lines of *CFB* do not show a discernible phenotype

To assess the function of *CFB*, mutant lines were investigated. Two T-DNA insertion lines were identified (SAIL_215_B11 and SALK_205373, henceforth called *cfb-1* and *cfb-2*, respectively). Both T-DNA insertions are located in the central region of the coding sequence downstream of the F-box-coding region (Supplementary Fig. S4). We were unable to detect any *CFB* transcript with primers on either side of the insertion sites, suggesting that these insertion mutants are null. None of the mutants showed an obvious phenotypic alteration in the vegetative and reproductive shoot when grown in the greenhouse. Additionally, investigation of root growth *in vitro* did not reveal any alteration in comparison to wild-type plants with respect to root length, lateral root development, and growth response to cytokinin (data not shown). The expression and induction by cytokinin of the primary cytokinin response genes *ARR5* and *ARR6* were unaltered in the *cfb-1* and *cfb-2* mutants in comparison to the wild type (data not shown).

### Overexpression of *CFB* causes the formation of white inflorescence stems

**Fig. 6.**
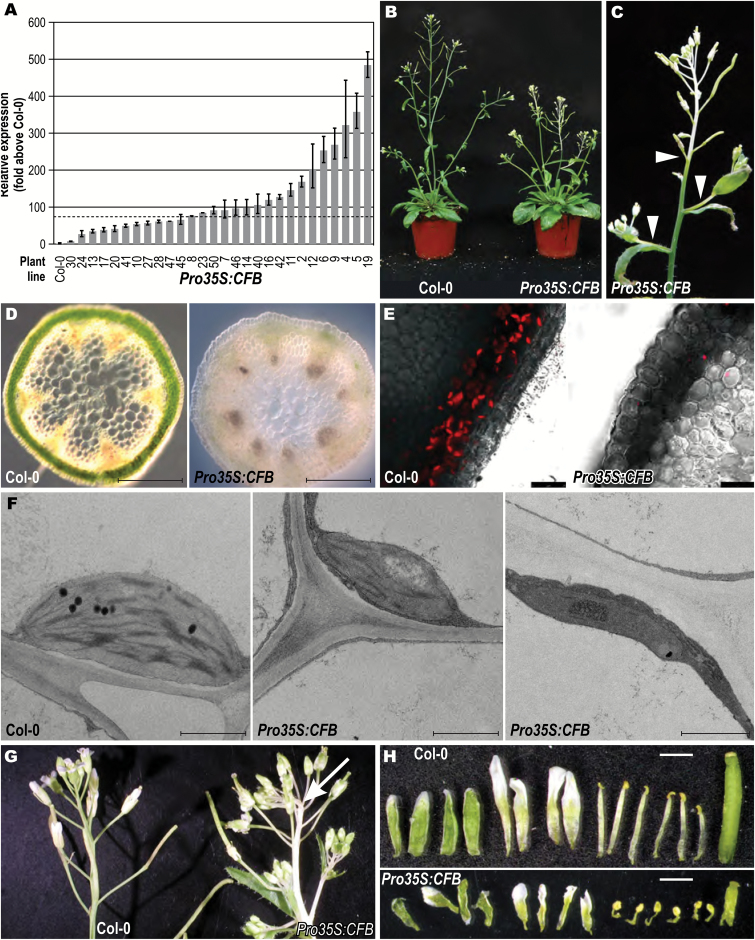
Phenotype of *CFB* overexpressing plants. (A) Relative *CFB* overexpression of selected primary transformants as revealed by qRT-PCR. The dashed line shows the expression level above which the white stem phenotype became apparent. (B) Phenotype of *Pro35S:CFB-19* in comparison to the wild type (Col-0), 16 days after sowing and grown under long-day conditions. (C) Inflorescence of the same plant as in B. Arrowheads mark the beginning of albinotic stem tissue. (D) Cross-section of the white inflorescence stem in line *Pro35S:CFB-19* and the corresponding region of the wild type. Bars=500 µm. (E) Fluorescence microscopy of cross-sections of a wild-type stem and the white stem of line *Pro35S:CFB-19*. Bars=25 µm. (F) Transmission electron microscopy of entire chloroplasts in wild type and in the white stem region of line *Pro35S:CFB-19*. Bars=500 nm. (G) Inflorescences of wild type and line *Pro35S:CFB-19*. The arrow points out the kinked growth of the main inflorescence stem. (H) Dissected flowers of wild type and line *Pro35S:CFB-19*. Sepals, petals, anthers, and gynoecium were separated from the floral axis and aligned to show the difference in organ size. Bars=1 mm.

To study the consequences of enhanced expression of the *CFB* gene, the full-length cDNA of *CFB* was stably expressed in Arabidopsis under the control of the *CaMV* 35S promoter. Plants with different transgene expression levels were identified by qRT-PCR among 94 independent transgenic lines. The increase in expression in these lines was between ~15-fold and ~500-fold; example lines are shown in Fig. 6A. Unless stated otherwise, all of the following data come from *Pro35S:CFB*-19, the line showing the strongest overexpression of *CFB*. Two other lines (*Pro35S:CFB*-23 and *Pro35S:CFB*-50) were also tested, with similar results (Supplementary Fig. S5).

Plants overexpressing *CFB* resembled wild-type plants during vegetative growth. After induction of flowering and elongation of the stem, plants exceeding a threshold of ~75-fold increased expression of *CFB* showed a characteristic phenotype comprising albinotic tissue at the distal end of the main inflorescence stem and the lateral branches (Fig. 6B, C, Supplementary Fig. S5). Lateral branches turned white in the internode proximal to the main stem (Fig. 6C). The percentage of albinotic stem tissue was positively correlated with the expression level of *CFB* (Fig. 6A, Supplementary Fig. S5C). The formation of albinotic stem tissue was accompanied by a shortening of the stem and the emergence of additional side branches from the rosette (Fig. 6B). The pedicels were white at the base and gradually turned green towards the flower. Cross-sections of the white part of the stem showed that the usually green chlorenchyma cells beneath the epidermis had almost no green pigmentation (Fig. 6D) and contained almost no chloroplasts (Fig. 6E, F). The few plastids present in this tissue were generally smaller than wild-type chloroplasts and contained, to a varying extent, fewer thylakoid membranes and fewer grana stacks (Fig. 6F). The stem tip remained white until senescence in the most strongly *CFB* overexpressing lines, while it became gradually greener over time in the less strongly overexpressing lines, indicating a dose-dependent effect of *CFB*.

To analyze whether the expression of chlorophyll biosynthesis genes or genes involved in chloroplast development is altered as a consequence of *CFB* overexpression, the level of such genes was analyzed in green and white stem sections of two strongly *CFB* overexpressing lines. Both CFB overexpressing lines showed essentially the same result. The transcript levels of almost all genes decreased in the white parts of the stem, while expression in the green parts of the stem of *CFB* overexpressing plants was mostly not altered, or only weakly altered, in comparison to wild-type plants (Supplementary Fig. S6). Notable exceptions are the genes *HEMA1*, *CHLH*, and *PSBR*, which showed lower transcript levels in the green parts of the inflorescence stems of *CFB* overexpressing lines.

**Fig. 7.**
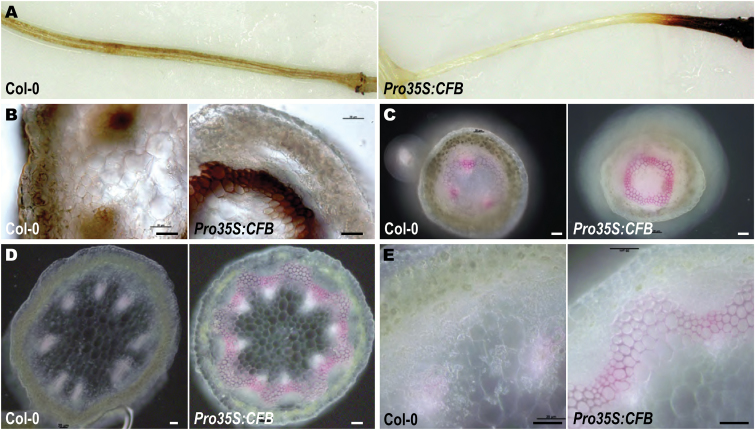
ROS (H_2_O_2_) accumulation and ectopic lignification in *CFB* overexpressing plants. (A) Magnified views of whole pedicels of wild-type and *CFB* overexpressing plants stained with DAB. (B) Light microscopic images of cross-sections of the DAB brown-stained region of pedicels of *CFB* overexpressing plants and the corresponding distal region of pedicels of the wild type (Col-0). (C) Light microscopic images of cross-sections of the green region of pedicels of *CFB* overexpressing plants and the corresponding distal region of pedicels of wild type plants stained with phloroglucinol to detect lignification. (D) Cross-sections of the white stem part of *CFB* overexpressing plants and the corresponding region of a wild-type stem, stained with phloroglucinol. (E), Images of the same sections as in D, at higher magnification. Bars=20 µm.

Plastid function can be impaired by reactive oxygen species (ROS) formed by the photosynthetic apparatus (Barber and Andersson, 1992; Aro *et al.*, 1993; Yamamoto *et al.*, 2008). We observed that the relative length of the albinotic stem parts decreased with decreasing day length (Supplementary Fig. S7), indicating a causal link between light dosage and the development of white stem sections. To examine whether light causes the formation of a greater amount of ROS in *CFB* overexpressing plants, leaves and shoots were stained with the H_2_O_2_ indicator DAB (Thordal-Christensen *et al.*, 1997; Snyrychová *et al.*, 2009). The staining patterns found in *Pro35S:CFB* transgenic plants and wild-type plants were similar in most tissues. In particular, staining was absent around the transition zone from green to white stem tissue. Only in the distal ends of the pedicels was DAB staining observed in *CFB* overexpressing plants but absent in the wild type (Fig. 7A). This section of the pedicels contained chloroplasts even in the most strongly *CFB* overexpressing lines. Cross-sections revealed that the staining was not in the chloroplasts of chlorenchyma cells, but in the cell walls of a parenchyma cell layer underneath (Fig. 7B). These cells had thickened cell walls, which were absent in the corresponding parenchyma cells of wild-type plants. Staining of these cell walls with phloroglucinol indicated that they were lignified, whereas lignification in the wild type was present only in the vascular bundles (Fig. 7C). Ectopic lignification and thickening of cell walls outside of the vascular bundles was also observed in sections of young stems of *CFB* overexpressing plants (Fig. 7D, E).

The length of the internodes of plants strongly overexpressing *CFB* was irregularly shortened and the inflorescence appeared to be more compact (Fig. 6G). With a penetrance of ~20%, the white stem sections were not growing straight, but were bending sharply at random points, indicating differential growth on opposing sides (Fig. 6G, arrowed).

The sepals and gynoecia of all flowers, including those growing on the white stem sections, were normally green (Fig. 6H). All floral organs were shorter than in the wild type (Fig. 6H), but they were fertile and produced green siliques of normal length filled with an ordinary amount of seeds. Siliques of strongly expressing *Pro35S:CFB* lines were often not straight, but were bent, kinked, or curled, indicating uncoordinated cellular growth (Fig. 6C).

Because *CFB* was most strongly expressed in the root, we examined whether overexpression of *CFB* had an effect on root growth. We could not detect any change in primary root elongation, the number of lateral roots, and the responsiveness of root growth to cytokinin in *CFB* overexpressing plants (data not shown).

### *CFB* overexpressing plants phenocopy the hypomorphic *cas1-1* allele and have a similar molecular phenotype

**Fig. 8.**
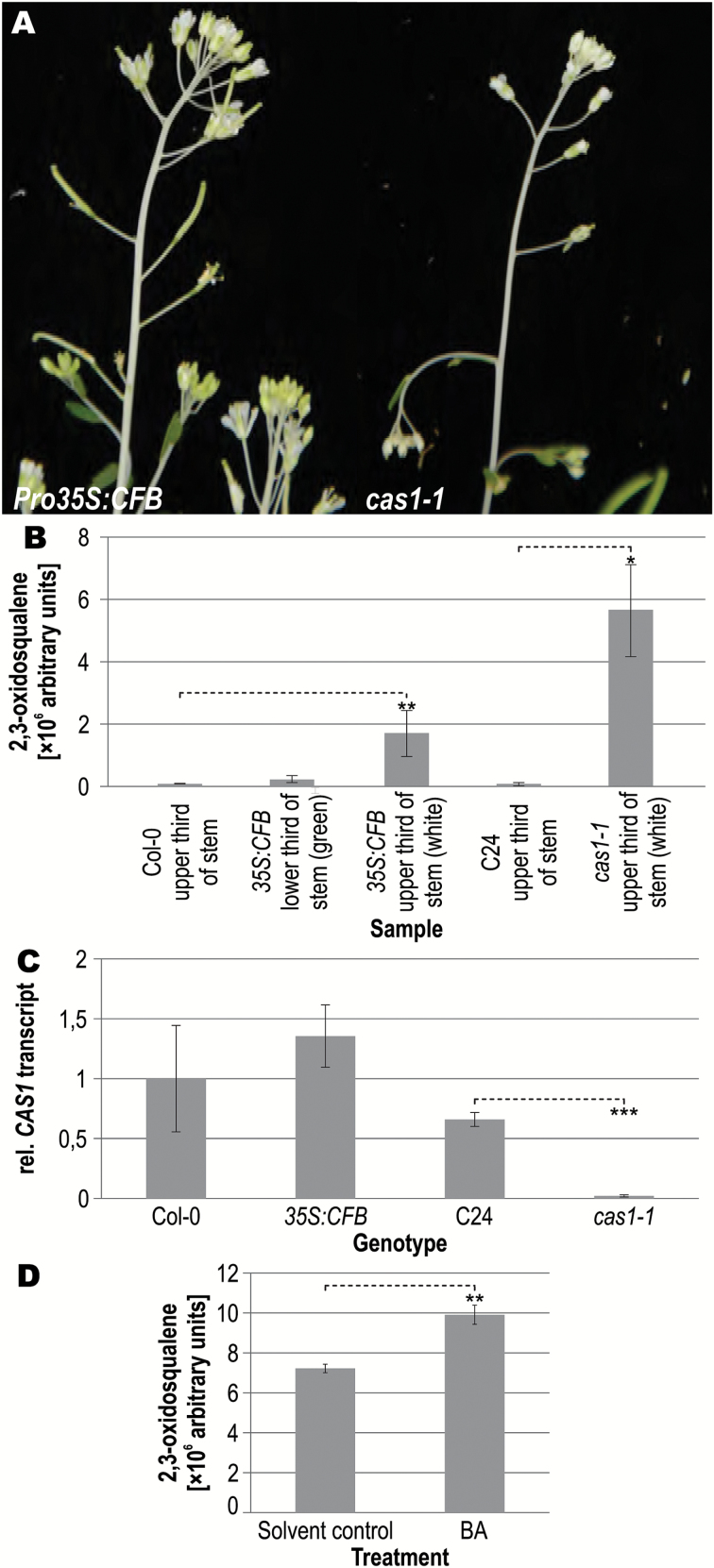
Phenotype of *CFB* overexpressing and *cas1-1* mutant plants. (A) Upper inflorescence of *CFB* overexpressing and *cas1-1* mutant plants. (B) Concentration of 2,3-oxidosqualene in wild-type (Col-0), *CFB* overexpressing, and *cas1-1* mutant plants. The content of 2,3-oxidosqualene was measured in inflorescence stem samples from the upper third of wild-type plants, the lower and upper thirds of *CFB* overexpressing plants, and the upper third of the stems of C24 plants and *cas1-1* mutant plants. Relative concentrations of metabolites of the sterol biosynthesis pathway downstream of 2,3-oxidosqualene are shown in Supplementary Fig. S8. Error bars=SD of two to four biological replicates. (C) Relative *CAS1* transcript levels in whole seedlings measured by qRT-PCR. The transcript level in Col-0 was set to a value of 1. Error bars=SD (n=3). (D) Concentration of 2,3-oxidosqualene in the upper third of cytokinin-induced inflorescence stems of *cas1-1* mutant plants. The content of 2,3-oxidosqualene was measured after spraying the plants with a solution of 5 µM 6-benzyladenine (BA) or a solvent control as described in the Materials and methods. Error bars=SD (n=3). Significance levels in comparison to the wild type (Student’s *t*-test): *P<0.05, **P<0.01, ***P<0.001.

The albinotic inflorescence stems of *CFB* overexpressing plants were strikingly similar to the phenotype of a mutant line named *cas1-1*, which is a partial loss-of-function mutant of the *CYCLOARTENOL SYNTHASE 1* gene (*CAS1*) (Babiychuk et al., 2008*a*, 2008*b*) (Fig. 8A, B). CAS1 catalyzes the cyclization of 2,3-oxidosqualene into cycloartenol, a key step in the plant sterol biosynthesis pathway. In *cas1-1* mutants, the concentration of 2,3-oxidosqualene, which is the substrate of CAS1, is elevated (Babiychuk *et al.*, 2008*a*, 2008*b*). Measurement of levels of metabolites of the sterol biosynthesis pathway in *CFB* overexpressing plants by GC-MS showed an accumulation of 2,3-oxidosqualene mainly in the white parts of the stems, where it was increased more than 20-fold in comparison with the corresponding wild-type tissue (Fig. 8B). The concentration of 2,3-oxidosqualene in the white stem tissue of *CFB* overexpressing plants was about one-third of that in *cas1-1* mutants. It is also noteworthy that the concentration of 2,3-oxidosqualene in the green parts of *CFB* overexpressing plants was only one-third of the concentration in the white parts. The concentrations of metabolites downstream of CAS1 were not altered, with the notable exception of sitosterol, which was significantly reduced by a factor of ~1.7 (Supplementary Fig. S8A). qRT-PCR data show that the transcript levels of *CAS1* were not altered in the albinotic stem parts of *CFB* overexpressing plants (Fig. 8C). Taking these findings together, *CFB* overexpression causes no alteration in *CAS1* transcript levels but results in accumulation of the CAS1 substrate, albeit to a lower level than in plants with altered *CAS1* expression or mutated CAS1 protein.

As *CFB* is a cytokinin-regulated gene and appears to be involved in regulating sterol metabolism, we attempted to assess whether cytokinin has an influence on the accumulation of the CAS1 substrate 2,3-oxidosqualene. However, 2,3-oxidosqualene was not detectable in the upper third of the shoots of wild-type plants, regardless of cytokinin treatment. We then reasoned that an influence of cytokinin would be most readily detectable in *cas1-1* mutant plants, which accumulate 2,3-oxidosqualene because of their strongly reduced CAS1 activity. Consequently, the relative amount of 2,3-oxidosqualene was measured in the upper third of the inflorescence stems of *cas1-1* mutant plants with and without cytokinin treatment (Fig. 8D). The results show that the amount of 2,3-oxidosqualene was further increased after cytokinin treatment of *cas1-1* mutant plants.

## Discussion

### Expression of the *CFB* gene

*CFB* was chosen for functional analysis because it was the highest-ranking uncharacterized cytokinin-regulated gene in a meta-analysis based on results obtained from CATMA microarrays (Brenner and Schmülling, 2015). Its regulation by cytokinin was confirmed by qRT-PCR analysis (Fig. 1A) as well as a transcriptomic analysis using RNA sequencing (Bhargava *et al.*, 2013). The rapid transcriptional response of *CFB* to cytokinin and the attenuated induction in type-B *ARR* double mutants strongly support the notion that regulation of *CFB* by cytokinin is achieved through the two-component signaling system. Its promoter contains several copies of the core cytokinin response motif [A,G]GAT[T,C] (CRM) (Ramireddy *et al.*, 2013).

Based on qRT-PCR and promoter-reporter gene analysis, the root was found to be the primary site of *CFB* expression, with the highest expression in the lateral root cap of the primary root and at the site of emerging lateral roots. Interestingly, induction of the *ProCFB:GFP-GUS* construct by externally applied cytokinin did not change the expression sites but only the expression level. In the lateral root cap, the expression is in accordance with the high cytokinin levels in these cells (Antoniadi *et al.*, 2015) and overlaps with that of the cytokinin signaling reporter genes *TCSn:GFP* and *ARR5:GUS* (Chang *et al.*, 2013; Zürcher *et al.*, 2013). These expression domains are thus consistent with a cytokinin-related function of *CFB*. In contrast, at the site of emerging lateral roots, *CFB* was expressed in a pattern that does not overlap with that of the cytokinin reporter genes, that is, as early as during the very first cell divisions and in later stages in a ring of cells around the developing lateral root primordium. This pattern is characteristic for *PIN6* and *CUC3*, which define the flanks of the lateral root primordia (Laplaze *et al.*, 2007). Taken together, the sites of *CFB* expression in the root and its cytokinin responsiveness suggest that *CFB* might participate in regulating the root system architecture, which is a well-known activity of cytokinin (Werner *et al.*, 2001, 2003; Riefler *et al.*, 2006; Laplaze *et al.*, 2007; Bielach *et al.*, 2012; Chang *et al.*, 2013, 2015). However, investigation of *cfb* mutants and *CFB* overexpressing plants did not reveal any discernible root phenotype; this could be due to experimental conditions and/or functional redundancy with *AT2G27310* and *AT2G36090,* the two close relatives of *CFB*.

### Structural and sequence relationship of CFB to other proteins

CFB belongs to a small subgroup of three proteins within subfamily E of the F-box superfamily (Gagne *et al.*, 2002). The close relationship between these proteins was found previously in a reciprocal BLAST analysis with the *Physcomitrella patens* SLY1 protein (Vandenbussche *et al.*, 2007). None of the three proteins has been characterized, and only *AT2G36090* was briefly mentioned as a down-regulated gene in habituated cell cultures (Pischke *et al.*, 2006). The three proteins of the CFB subgroup differ from any other F-box protein in their domain structure. Apart from the F-box and transmembrane domains, they do not contain any known additional domain; in particular, they have no known protein–protein interaction domain. Therefore, the three proteins of the CFB group cannot be assigned to any known structural group of the F-box superfamily of proteins, and no role can be deduced for them on the basis of sequence similarity.

### CFB is a structural constituent of an E3 ubiquitin ligase complex

An intact F-box is required for the association of F-box proteins with SKP1/ASK1 (Deshaies, 1999). The F-box-dependent interaction of CFB with ASK1 proves that CFB is part of an E3 ubiquitin ligase of the SCF family. Therefore, it is expected that CFB interacts with at least one partner that will be marked by polyubiquitination for degradation through the proteasome. The substrate specificity of F-box proteins is mediated by sequence motifs, which are often located C-terminal to the F-box domain (Patton *et al.*, 1998). The absence of any known interaction domain apart from the F-box domain suggests that an as yet unknown domain or motif mediates interaction between CFB and its so far unknown partner(s). It is probable that one of the conserved sequence regions C-terminal of the F-box domain may function as a novel protein–protein interaction domain. Motifs within these domains that are potentially relevant for substrate recognition are the highly conserved sequences LSWI(L/V)IDPXXKRAA and ELISAVD. Among the F-box proteins, both motifs occur exclusively in the CFB subgroup proteins. Identification of one or several interaction partners of CFB and its sequence-related proteins would yield information about the functional context of these proteins. Regarding the lack of a mutant phenotype, it should be considered that loss of function of only a small number of F-box proteins causes a discernible phenotype; most phenotypes might be subtle, context-dependent, or masked by functional redundancy. Notably, the two *CFB* homologs *AT2G27310* and *AT2G36090* are also expressed in the root (Winter *et al.*, 2007), making the investigation of higher-order mutants worthwhile.

### The subcellular localization of CFB depends on two functional domains

F-box proteins have generally been found to be localized in various cellular compartments, excluding mitochondria and plastids, but including the cytoplasm and the nucleus (Kuroda *et al.*, 2012). Consistent with the function of CFB as a facultative constituent of an E3 ubiquitin ligase complex, which has also been shown to be localized in these two cellular compartments (Farrás *et al.*, 2001; Shen *et al.*, 2002), GFP-CFB fusion proteins were localized in the cytoplasm and nucleus. Furthermore, the protein appeared to be localized to the plasma membrane. Localization at the plasma membrane was dependent on the annotated transmembrane domain. This observation was supported by immunodetection analysis of the CFB-GFP fusion protein in Arabidopsis seedlings. Full-length CFB protein and CFB without the N-terminal F-box domain were enriched in the purified microsomal fraction containing membrane-bound proteins, but this was not the case for CFB lacking the predicted C-terminal transmembrane domain. It could be that the mode of action of CFB is similar to that of certain receptors and other signaling proteins, which are activated by being cleaved off from their transmembrane domains (Johnson *et al.*, 2008; Chalaris *et al.*, 2011; Chen and Hung, 2015). The nuclear localization signal appears to be located near the F-box domain at the N-terminal end, as truncated versions of CFB lacking this domain were excluded from the nucleus. However, none of the known nuclear localization signals was identified with certainty in the F-box domain of CFB. A possible mechanism for nuclear retention of CFB could be based on the interaction of the F-box domain of CFB with ASK1 of nuclear-localized E3 ligase complexes (Farrás *et al.*, 2001). The functional importance of the subcellular localization was demonstrated by the observation that transgenic lines overexpressing N- or C-terminally truncated versions of CFB never showed the characteristic phenotype of plants overexpressing a gene encoding a full-length CFB-GFP fusion protein.

### The phenotype of *CFB* overexpressing plants suggests an impact of CFB on sterol biosynthesis, influencing chloroplast development and function

Plants strongly overexpressing *CFB* showed pleiotropic phenotypic alterations, which became more severe with increasing *CFB* gene expression. The most obvious anomaly was the presence of only few and partially abnormal chloroplasts in the upper inflorescence stem, resulting in low chlorophyll content and the formation of white stems. The fact that tissues growing on the albinotic stems, such as siliques, were green, and that under lower expression of CFB albinotic stems were able to slowly become green, indicates that there was no complete loss of plastids, but rather a failure to develop mature chloroplasts.

As the transition from proplastids to mature chloroplasts is a highly complex process, many causes that can prevent plastids from developing into mature chloroplasts must be considered. Many of the mutations that cause failure to develop chloroplasts are lethal at very early stages of plant development. Viable forms are albinotic only in part of the tissue; for example, they may have variegated leaves. Genes affected in albino or variegated mutants have a wide variety of functions, such as chlorophyll biosynthesis (Ruppel *et al.*, 2013), repair of photooxidative damage (Yu *et al.*, 2007), maintenance of mitochondrial genome integrity (Sakamoto, 2003), or sterol biosynthesis (Kim *et al.*, 2010; Lu *et al.*, 2014). Investigation of the expression of genes involved in chlorophyll biosynthesis and chloroplast development did not reveal a blockage at a particular point of the pathway, reflecting only the absence of chloroplasts. We cannot rule out that this pathway is disturbed at a process other than transcription.

The intensity of the white inflorescence stem phenotype was positively correlated with light dosage, suggesting increased photodamage. The prime reasons for photodamage are ROS, generated by, for instance, photosystem I (Mehler, 1951). As we were unable to detect ROS in the chloroplast-containing cells by DAB staining, particularly in the transition zone from green to white tissue, we cannot substantiate this idea. Alternatively, the failure of juvenile plastids to propagate and develop into mature chloroplasts might be due to other reasons, such as hampered pigment or membrane biosynthesis or lack of a developmental factor.

As CFB is an F-box protein and as such is likely involved in targeting specific proteins for proteasomal degradation, the white stem phenotype of *CFB* overexpressing plants suggests that one or several of the CFB target proteins are required to promote the development of plastids into chloroplasts. According to this hypothesis, overexpression of *CFB* would generate a dominant-negative phenotype by targeting a larger amount of its target proteins for degradation. Little is known about the role of the ubiquitin–proteasome pathway in chloroplast development. Recently, a RING-type E3 ubiquitin ligase was characterized that targets the protein transport complex at the outer plastid envelope (TOC) for degradation, thereby facilitating the reorganization of the chloroplast import machinery in response to stresses (Ling *et al.*, 2012; Ling and Jarvis, 2015). It is possible that the SCF^CFB^ E3 ligase is able to target another functionally relevant component of chloroplast development, causing its arrest or retardation. The interference of ectopically expressed *CFB* with chloroplast development and its predominant expression in the root would be consistent with a role in suppressing the formation of chloroplasts in the root, either directly or indirectly. Additional experiments are required to substantiate the function(s) of CFB.

The *CFB* overexpressing plants phenocopy the albinotic inflorescence stem tips of the hypomorphic *cas1-1* mutant (Babiychuk *et al.*, 2008*a*, 2008*b*), which is defective in the *CAS1* gene encoding a key enzyme in plant sterol biosynthesis. CAS1 protein converts 2,3-oxidosqualene to cycloartenol in the sterol biosynthesis pathway. At the molecular level, *CFB* overexpressing plants accumulate 2,3-oxidosqualene, like the *cas1-1* mutant, which has residual CAS1 enzyme activity. This suggests that in *CFB* overexpressing plants the sterol biosynthesis pathway is impaired in a way similar to that in the *cas1-1* mutant. Transcript levels of *CAS1* are unaltered in *CFB* overexpressing plants. This raised the idea that CFB may reduce CAS1 activity by targeting either the CAS1 protein directly or a factor that promotes its activity for ubiquitination and subsequent proteasomal degradation. Alternatively, a mechanism independent of protein degradation can be conceived of, similar to the direct regulation of the activity of the squalene synthase Erg9 by the F-box protein Pof14 in yeast (Tafforeau *et al.*, 2006). Consistent with both options is the finding that cytokinin treatment of *cas1-1* mutant plants led to a further increase in 2,3-oxidosqualene levels in the white stem tissue. The molecular details of this apparent regulatory link between cytokinin and sterol metabolism, the role of CFB, and the tissues in which it is functionally relevant will be addressed in the future.

The mechanism by which the *cas1-1* mutation causes the albinotic stem tip phenotype is unclear. It may be speculated that there is a lack of an essential metabolite for chloroplast biogenesis owing to the blockage of the sterol biosynthesis pathway. Consistently, impairment of sterol biosynthesis at different points of the pathway may lead to defects in chloroplast development (Kim *et al.*, 2010; Lu *et al.*, 2014). Toxicity of the accumulating 2,3-oxidosqualene for plastid biogenesis during certain developmental phases also cannot be excluded.

In *CFB* overexpressing plants, cells in the intervascular space prematurely develop thickened and lignified cell walls, which normally happens only after secondary growth has started, by activation of a ring of cambial cells (Sanchez *et al.*, 2012). In this context, *CFB* action would appear to promote an advanced developmental stage causing premature differentiation. Interestingly, mutants of the sterol biosynthesis pathway have been found to ectopically accumulate lignin (Schrick *et al.*, 2004), corroborating the idea that defective sterol biosynthesis is a major cause of the phenotype of *CFB* overexpressing plants.

## Supplementary data

Supplementary data are available at *JXB* online.

Fig. S1. Histochemical staining of *CFB* promoter induction by cytokinin in two independent transgenic lines carrying a *ProCFB:GFP-GUS* reporter gene.

Fig. S2. Multiple sequence alignment of Arabidopsis CFB, AT2G27310, and AT2G36090 and orthologs of other dicotyledonous plant species.

Fig. S3. Phenotype of plants overexpressing a *CFB-GFP* fusion gene.

Fig. S4. Analysis of the *CFB* transcript in *cfb-1* and *cfb-2* mutants.

Fig. S5. Comparison of independent *CFB* overexpressing lines to the reference line *Pro35S:CFB*-19 and wild type.

Fig. S6. Expression of chlorophyll biosynthesis and other chloroplast-related genes in green and white stem sections of two independent *CFB* overexpressing lines.

Fig. S7. Formation of the albinotic stem tip of *CFB* overexpressing plants grown under long-day (16h light/8h dark) and short-day (8h light/16h dark) conditions.

Fig. S8. Relative concentrations of sterol metabolites in different genotypes and tissues.

Table S1. Cloning procedures and PCR primers used in this study.

Table S2. qRT-PCR and sequencing primers.

## Supplementary Material

supplementary_figures_S1_S8

supplementary_tables_S1_S2
