# Supplementary material for: Analysis of CFB, a cytokinin-responsive gene of Arabidopsis thaliana encoding a novel F-box protein regulating sterol biosynthesis
Source: J Exp Bot. 2017 May 12;68(11):2769–85. doi: 10.1093/jxb/erx146 (PMC5853388; doi:10.1093/jxb/erx146)
Supplement: supplementary_figures_S1_S8 [file erx146_suppl_supplementary_figures_s1_s8.pdf]

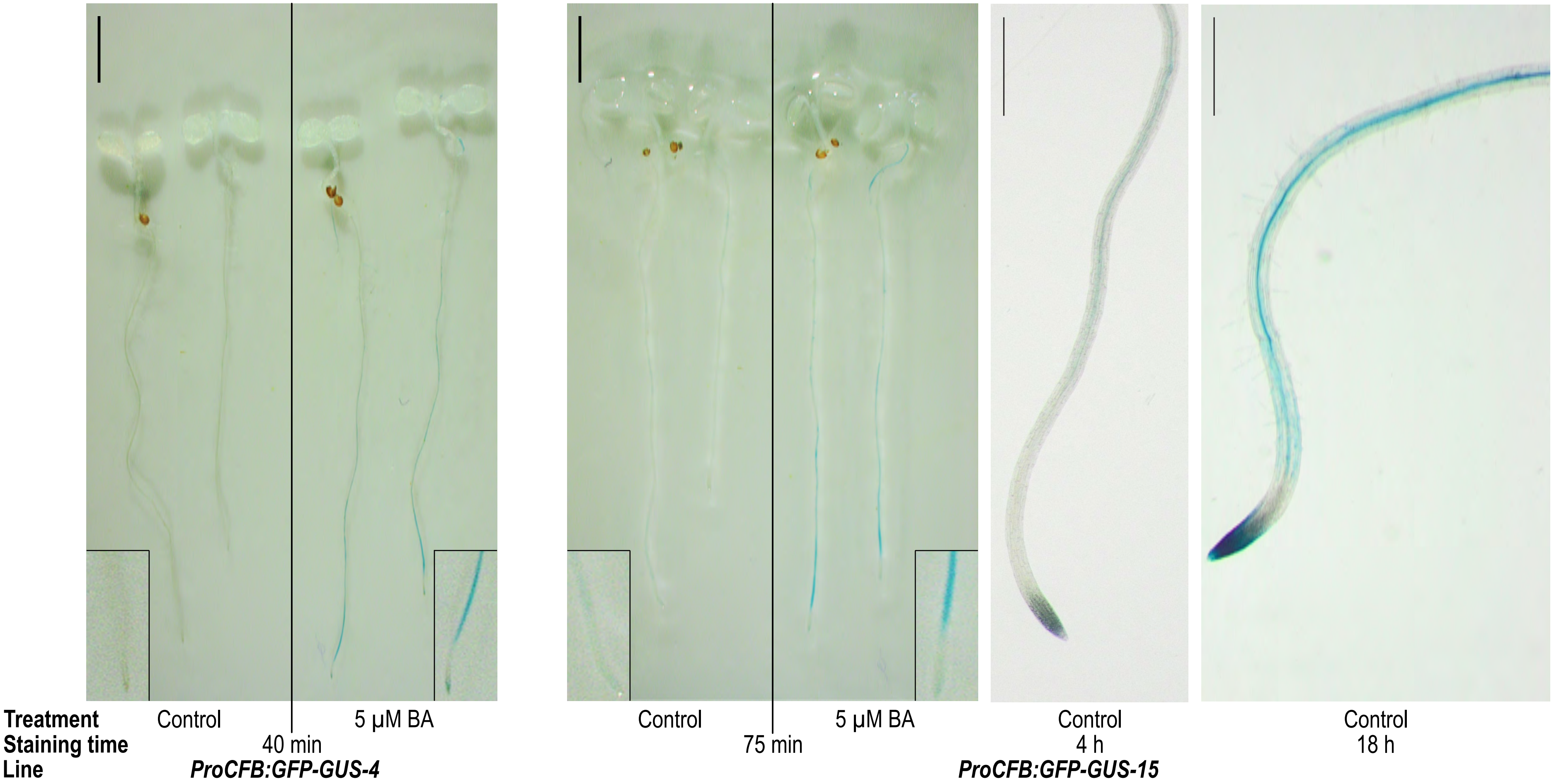

Figure S1. Histochemical staining of *CFB* promoter induction by cytokinin in two independent transgenic lines carrying a *ProCFB:GFP-GUS* reporter gene. 6-day-old seedlings were treated with 5 µM of the synthetic cytokinin 6-benzyladenine (BA) or a solvent control for the times indicated. Subsequently, the seedlings were stained according to Jefferson et al. (1987) for the times indicated, inspected and photographed as described in Materials and methods. The insets show magnifications of root tips. Scale bar = 5 mm.

```
                               1                                                                                                                       130
  CFB [Arabidopsis thaliana]                                                                MLS SSSSS-TVEQ PSR-GGSPGI NAVSSDVLRS NILTRLDGSS LAALSCTCSN LNSFCS-DES LWRQQCSATW
          Arabidopsis lyrata                                                                MLS SSSSL-TVEQ PSR-TGSSGI NAVSSDVLRS NILTRLDGAS LAALSCTCSN LNSFCS-DES LWMQQCSATW
            Capsella rubella                                                              MILSS SSSST-MMEQ PSSYLESSGI NAVSSDVLRS NILTRLDGAS LAALSCTCSN LHSFSS-EES LWKQQCSVTW
             Camelina sativa                                                                  M LSSSS-MVEQ PSR-IKISGI HAVSSDVLRS NILTRLDGAS LAALSCTCSN LHSFSS-EDS LWKKQCSLTW
        Tarenaya hassleriana                                                              MLSSS YSHPLPTAEH PPRGGVGTTI SAVPHDVVRS HIIPCLDGAS VAALSSASVE LHSLCT-DEH IWRDKCIATW
               Arabis alpina                                                                     MLPSTAEQ PSH-TGSCDI NAVSSDVILT HILTRLDGAS LAAVSCTCSH LHSFSS-EES LWKKTCTATW
                   AT2G27310                                                                           MA TVDVINGDSI STLHSDIIQT QILTRLDGPT LASTATTSSY LQTLCT-EEK LWQELSIDTW
             Ziziphus jujuba                                                               MPSP HPTDS---TA NVDDDGAISI SAVHPDIIQA HILTRLDGPT LASAGCASSH LHALST-QDK LWRDICTSTW
             Morus notabilis                                                                 MA TSDNM---AV GGGSGGGTTI SAIHPDIIQA HILARLDGPT LAAAGCASSE LHALSA-DEN LWRDICATMW
          Eucalyptus grandis                                                               MPSP STPLAGADAA PATAHAATVL TSIHPDVIRT HILTRLDGPA LVAATSASSQ LHSLSSSNDC LWRDVCIAAW
              Vitis vinifera                                                                                MDSPSTTI TSVHPDIIQT HILTRLDGPT LAAAGAASSQ LHALAT-VDK LWRDICTSTW
          Gossypium hirsutum                                                                     MNSTTSLPLH QGG---NTPI SALHPDIFQS HILALLDGPS LASLACVSSQ LHALST-HDI LWFNICSSTW
             Theobroma cacao                                                                     MKTITSTTVD QGGYASNETI STVHPDILHS HILTRLDGPS LAALACSSSH LHALSS-EEN LWQNICSSTW
              Fragaria vesca   MEAVVDSLPP KTKDHQLPQE YYITKPLQPS LCHHSHPHFK LQLDLRLTNV QLELELEEEE QYFDREMASF LVEQGGDAS I SALHSDIITS HILTRLDGPT LASAACTSSQ LRAFSA-DQT LWKDICATTW
             Malus domestica                                                                      MSFSAVTTL AVDQGDRPSI SVVHPDIITA HILTRLDGQT LAAASCASSE LHSFSA-KEK LWRDASIATW
              Prunus persica                                                                      MGFSA-TSP TVDQGDGASI SAVHPDIIMA HILTRLDGQT LASASCASSQ LHAFST-EGK LWRDICSATW
            Ricinus communis                                                                         MVPPSS TLHHHENGTI STVHHDILQT HILTRLDGPT LASLSCASSD FHTLSS-QDK LWQNICSFTW
             Jatropha curcas                                                                 MV AFPSSTPPPP TVDPIGDGTI SAVHHDILQN HILNRLDGPT LAAVACASSE LHGLST-EDK LWQKICIDTW
          Manihot esculenta                                                                           MAPPS NVD--GTVSI SSLHLDVLHH HILSRLDGPS LAALSCTSSE LNALSS-YDK LWQQICTSTW
         Populus trichocarpa                                                                 MV ALSTPPPQQP SSTTTQDGTI STLHPDILQT HILTLLDGPT LAATACASSE LYALST-EDK LWRNICTSSW
            Nelumbo nucifera                                                             MAISAC KSSAKTMTTD EKECGATTTI SALHPDVVQA HILTRLDGPS LASVCCASSQ LHALSN-DES LWRDMCHSTW
          Citrus clementina                                                                           MQMAP PSFDQNTARI SAVHPDIIET HILTRLDGPT LASASCVSSQ LHSLAS-DES LWTNICHSTW
                Cucumis melo                                                                     MPSPPPSFSF HHRSSAEDAI AALHSDILQT HILTRLDGAA LTSTASTSSR FRRLSS-EDQ LWRRVCSTSW
               Daucus carota                                                           MLSLLHKD SNMVSHISDH QQERNNSSSI LDIHSDIFSN HILTRLDGSS LVSTGSASSE LNKFCK-DEN LWKNICNSTW
    Dorcoceras hygrometricum                                                                      MSSYTSAAA AEDHGGGTPI TAIHSDIVRS HILNRLDGPT LASTSCASSR LFSLCN-DDH LWREICSSTW
           Arachis duranensis                                                                                MDIDMI TNLHSDIINS HILTRLDGPT LASAAASSSH LRRLCT-QHH LWTTICTSTW
               Vigna radiata                                                                           MPPM DATSSSSSDI TTLHPDIIRS HILNRLDGPT LASASSSTLH LRRLCS-DHN LWRTICAATW
          Phaseolus vulgaris                                                                      MDTTSFSSS SSSSSSSSNI TTLHPDIIQS HILNRLDAPT LASASSTTLH LRRLCT-DQH LWRNICAATW
                 Glycine max                                                                           MTPA MDKTSSSSDI TTVHPDIFQS HILNRLDGPT LASAASATSH LRGLCT-EHH LWRNICAATW
          Cynara cardunculus                                                                                    MAI ADIHQDIIKT HILTRLDGQT LSAAACVSLQ LQSLCS-DDK LWSGICSANW
             Sesamum indicum                                          MEFKS SSKGVCATFH SPLITTASTE PKNENEMPLA TADQGGANGL SALHPDIVRS HVLSRLDGPA LGSAACCSTT LRQLSS-DEN LWSNICHSMW
        Erythranthe guttata                                                                        MQHGATAT TTEHGGAAGF SALHPDIIKS HVLTRLDGPA LGSAACCSAT LRHISD-DGK LWSDICHSTW
              Beta vulgaris                                                                       MSSSPPMST TTTTTLTTAF SLLPDDVIRA HILPRLDGCS LDAVGCATPE LSSLSS-DHQ LWSHICHSTW
          Medicago truncatula                                                                        MICISG ESEAEETTTI AAVHSDIIQT HILTRLDGPA LASAASTCSQ FNSLCT-NEH LWANMCHSTW
             Cicer arietinum                                                                 M PKATMSNISG NPITAETTTI SAFHSDIIQT NILTRLDGSA LASVASTCSR FHYLCS-TEH LWENACYSTW
              Cajanus cajan                                                                          MVCTTD EASCG-ASAI SAVHPDVIQT HILTRLHGPA LACVASTCSE LNALSA-HEP LWAEICRATW
           Nicotiana tabacum                                                                      MSSCSTTTT TSDDGGRTAI TAVHPDIIQI LILTKLDGPT LISTSSASSQ LKNLCS-DDI LWQKICNSYW
                   AT2G36090                                                                        MANSSSF SPSTTVTDLI STVHDDIIES HILTRLDGAT LASVSCASSH LHHLAS-NEI LWSKICRSTW
                   Consensus                                                             ...... .......... .........i sa.h.D!i.. hILtrLDGpt Las..c.ss. l..ls. ... LW..iC.stW
           Conserved regions
```

Figure S2. Multiple sequence alignment of Arabidopsis CFB, AT2G27310, and AT2G36090 and orthologs of other dicotyledoneous plant species. The alignment was carried out using MultAlin (http://multalin.toulouse.inra.fr/multalin/) with the standard settings (Corpet, 1988). The colored lines correspond to the conserved regions shown in Fig. 2A. Red and blue amino acid symbols denote highly (≥ 90 %), and less (≥ 50 % and < 90%) conserved residues, respectively. Symbols for amino acids other than letters in the consensus sequence: ! = I or V; $ = L or M; % = F or Y; # = N, D, Q, E, B, or Z.

```
                             131                                                                                                                                260
CFB [Arabidopsis thaliana]  PST-SDTRVQ SIISTFPDG- HRTFFSDSFP FLEH------ ---DVGIN-L PPSVDTSELI SAVD------ ---IFYKDDV IFSRVHVTET V---SGWFLC SPMRVDLVEP -KELIPTRVL VTDQCKD-DT
      Arabidopsis lyrata  PST-LDTRVQ SIISTFPAG- HRQFFSDSFP FLEH------ ---DGVIN-L PPSVDMTELI SAVDMTELIS AVDIFYKDKV IFSRVHVTET V---SGWFLC SPMRVDLVEP -KELISTKVS VVNQWED-DT
        Capsella rubella  PST-LDARVQ SIISTFPAG- HRTFFSDSFP FLGQ------ ---DGVIN-L PPSVDTTELI SAVD------ ---IFYKDEV IFSKVHVTET V---SGWFLC SPMRVDLVEP -KEMVSTKVS VVEQWED-DT
         Camelina sativa  PST-LDARVQ SIIATFPVG- HRTFFSDSFP FLER------ ---NGVMNNL PSSVDTTELI SAVD------ ---IFYKDQV IFSKVHVTET V---SGWFLC SPMRIDLVEP -KELISTRVS LVNQWED-DT
     Tarenaya hassleriana  RSF-LDPRVQ RVISTLPSG- HRSFFSDSYT HLGL------ ---RRGLVDL PPSVATKELI SAVD------ ---IYYKGEV IFSKVQETET V---SGWFLC SPFRVDLLDP -KESVSTRIP YNDQWDE-ET
            Arabis alpina  PST-SDTRVQ SIISTFPSG- HRSFFSDCFP FLG------- ----GEIDLT PPSVDTTELI SAVD------ ---IHYKDEV IFSKVQATET I---SGWFLC SPMRVDLVEP -KELITTRVS LIDQWEN-DT
                AT2G27310  PSI-NDPRVV QAISSFPSG- YRSFFADSYP FTEH------ ------TWQS EKHDPPTGLI SAVD------ ---LYYRGEI IYSKVQEMET EKGKSGWFLS SPFRVDILDP -KESVQTRIR YPGGDYE--A
          Ziziphus jujuba  PSI-NDPRVQ RLISSFPSG- HRSFFSDSFP LLDP-----S SFCRFGHG-- RPLSSTSELI SAVD------ ---IFYKDSL IFSKVQEIET L---SGWFLC SPFRVDLLDP -KESAPTPIQ HVAGEDD-GA
          Morus notabilis  PSV-ANPALR RLISTFPAG- HRSFFSDSFP VLDR-----V PPQTFNPD-- PPFLS-SGLI SAVD------ ---IFYKDKL IFSKVKETET L---TEWFHC SPFLVDLLDP -KDSVQTPIQ RAA-VDD-GA
       Eucalyptus grandis  PSV-RDPRVA EAIATFPSG- HRSFFSDSYP VLHH-----H KLRRRRSP-- PPPMA-ECLI SAVD------ ---VHYEGEA IFSKVHETET V---SGWFEC SPFRVDLLDL -KESVPTRIQ KPGGTPD-EA
           Vitis vinifera  PSL-NDPTLQ ALVSAFPGG- HRSFFSDSYP LLAHPLDQYH GQSRKIPP-- TTSSPTLELI SAVD------ ---IHYKNSL IFSKVEATET I---TGWFQC SPFRVDLLDP -KDTVPTPVQ YSV-DGD-YK
       Gossypium hirsutum  PSL-NHPRLQ QIISTFPSG- HRSFFSDSFP FPDL------ -QPLKLDV-- NSCTLPTELI FAVD------ ---VYYQNQI IYSKVEELDT S---SSWFLC SPFRVDLLDP -KDSASTPVR YLGGSQD-EA
          Theobroma cacao  PSV-NHPRLQ KIISAFPSG- HRSFFSDAFP FLDL------ -QPLKLNV-- NSLTLPTELI SAVD------ ---ISYRNKI IYTKVEEMET S---SSWFLC SPFRVDLLDP -KDSAPTPIK YFGGSNKVDT
          Fragaria vesca  PSV-SDKRVH DIISTFPSG- HRSFFSDSFP LLDH-----S ACSSDTSS-- RLVIPASELI SAVD------ ---IFYKGEL VFSKVHETET E---TGWILC SPFRIDLLDP -KETVPTPIR HIGETN---E
          Malus domestica  PSI-TDPRVN DLISTFPGG- HRFFFSDSFP LLDH-----S PSQFNFS--- PSSP-TAELI SAVD------ ---IFYKDQL IFSKVQESET E---SGWFLC SPFRVDLLDR -KETVPTPIQ HVGEDQ---K
          Prunus persica  PSV-ADPRVD DLISTFPAG- HRSFFSDSFP LLDN-----F PSRFDLSR-- PSSPPTTELI SAVD------ ---IFYKDQL IFSKVQESET E---SGWFLC SPFRVDLLDP -KETVPTPIR HVGEDQ---A
        Ricinus communis  PST-NDPLLS SVISSFPSG- HRSFFSDSFP LLRH-----H RPTCRTLP-- TPFPSTTELI SAVD------ ---IYYRNVP IFSKVEKTET V---SRWFLC SPFRIDLLEP -KEFVPTWIQ -KAGEKD--S
         Jatropha curcas  PSI-NDPLTS SIISTFPSG- HRSFFSDSYP LLHH-----H RHSSHILE-- RPSPETTELI SAVD------ ---IFYQNFP IFSKVEETET E---SGWFMS SPFRVDLIEP NKDFVPSGIP -QASEND--S
       Manihot esculenta  PSV-NHPLVT HAVSTFPSA- HRSLFSDSYP LLLH------ RHSCHDLD-- RPFPATRELI SAVD------ ---IYYQNVP IFSTVEATET V---TGWFLS SPFRVDLVGP -EEFIPTRIQ -QIGEKN--S
      Populus trichocarpa  PSI-NDPTVS SIISTFSSG- HRSFFSDSYP LLHH-----H HHSSSFLT-- T---STEYLV SAVD------ ---IYYKDVA IFSKVEKNET L---TDWFKC SPFRVDMLEP -KEFVQTLIQ YQTGEKD--S
         Nelumbo nucifera  PST-DTPRIR DIVSSFPAG- ARSFFSDSFS LLVSP---HD QAPPSNLN-- PPVS-PPEII SAVD------ ---IHYRGNL LFSKAHETET L---SGWFRC SPFRVDLLDP -KEVVPTPIT ISDG------
       Citrus clememtina  PST-NTPRLR HVISSFPYG- PRSFFSDSFT N--PD---QT PASTSSSS-- PSLNRPSEII SAVD------ ---VYYKNKL IFSRVVETET A---SGWFNC SPFRVDLLDP -KDSVPTRIP HPDNED----
            Cucumis melo  PSI-THPKLQ QLISTFPSK- HQSFFSDVFP VLDC------ CSLRCDLD-- YRYSSTAELI SAVD------ ---IHYKNKL LFSKVHSIET E---TNWFLC SPFRVDLIDP -KDSIPSPIR RSEKYED---
           Daucus carota  PST-DDRRLL DIIAAFPAG- YRSFFSDCYP SLDHSAKPKV QLVDREIEGR QSLSKISELI SAVD------ ---IRYDNNL IYSKVQSTET S---TDWFLS SPFRVDLLDP -KETIPTPVP VLEGNKD--L
  Dorcoceras hygrometricum  PST-TDPRVR AVISGFSSG- YRSLYSDAFP SVAH-----Q RSAGRRTKKR SLPSGTSELI SAVD------ ---IYCDDKL IYSKVMVTET H---SGWFMC SPFRLDLLDP -KETVPVPLI FDGEDGN---
       Arachis duranensis  PSL-THPLAA HLISTLPNQ- HRSIFSDSFP SLHP------ -SALHN-NQK PPPSPSPELI SAVD------ ---IYYNGKP VFSRVHRTET Q---KGWFLC SPLWIDILEP -NETVPTPLK FAKSEDD-DD
           Vigna radiata  PSL-NQPHAS ALIDTFPAA- HRSVFSDSFP SIQY------ -SPP---PNP IPPRLSPEFV SAVD------ ---LYYKGRP VFSRVITTET H---KGWFLS SPLWVDLLDP -NEVVPTPLI FAKCDEE-SS
      Phaseolus vulgaris  PSL-NQPRAA DLIATFPAA- HRSVFSDSFP SIHH------ -SPS---PHP TPTRPPPELV SAVD------ ---LYYKGKP VFSRVITTDT Q---KGWFLS SPLWVDLLDP -NEVVPTPLI FAKCEEE-SS
             Glycine max  PSL-NDPLAA ATIATFPAT- HRSIFSDSFP SLHH------ -SPPNPIPTQ PPTPPPPELI SAVD------ ---IYYKGKP VFSRVIRTET H---KGWFLC SPLWVDLLDP -TEVVPTPLK FAQTNDE-IE
       Cynara cardunculus  PST-DDPLVR QAISNFPSG- HRSFFSDSYP FPSH------ ---RLTTTTT SPSAPTSQII SAVD------ ---LRYHDEL VFSKVESTNI TH--SDWFRS SPFRIDLLEP -KELVPSAVK FSGDDH---V
          Sesamum indicum  PST-TSPQLS HLISTFPGGG PRAFFSHAFP VLA------- -ADRNLTRSS S--NPPSELI SAVD------ ---IRYKDNL IFTKVQETET V---SGWFRC SPFRIDLLEP -KDVVPTP-- ----------
      Erythranthe guttata  PSTASSPRLS HLISNFPDGG ARAFFSHAFP VVA------- -ADGGSVRPP S--NPPAELI SAVD------ ---IFYKGKL IFSKVQETET V---TGWFRC SPFRIDLLDP -KDVVPTRVK HPEGDG----
            Beta vulgaris  PSTAT-PRIR HLISSFPDG- ARSFFSMSFP LLS------- -PNPHPPLPS SCCSLPDQLI SAVD------ ---IFYRGKC MFSNVQETET V---TGWFRC SPFRIDLLTP -KESVPTPIR RPSHDD----
      Medicago truncatula  PST-NTERVQ QVISNFPNG- FRSFFADSFS SHHQ------ -RDMTLT--- -NHDETLSLI SAVD------ ---IFHREGL VFSKVVETET V---TGWFRC SPFRVDLLDT -KDVIKYPVV GDENNV----
          Cicer arietinum  PST-NTPCVR HAISTFPNG- SRSFFADCFS SHYL------ -ATTTIETAS MNLDRTPRLI SAVD------ ---IFHRKRL VFSKVVETET V---TGWFRC SPFRVDMLEI -KDVIKCPVQ D---------
            Cajanus cajan  PST-SAPRVR HVISTFPRG- SRSFVSDSLA SFSA------ -RGWREA--- -SADRAPELI SAVD------ ---VFFEGRA VLSRVVETET E---SGWFRC SPFRVDVLDP -KEAAATAVE YPRSEE----
        Nicotiana tabacum  PSS-SNPLVQ NAISTFPSG- HRSFFSDSFP AILH------ HNNTNICRTE YRNFLTSELI SAVD------ ---IHFEENL LYSKVVKTET K---SGWFMT SPFRVDLLGH -KETVTTPVK FDGDDG---I
                AT2G36090  PSC------- -------SGG SRSFFSDAYS MVE------- ----TAGTVS DLDRPFPELI SAVD------ ---LHYRGKL IFSRVVKTET T---TAWFKS SPLRIDLVDT -KDTVATPIK RRQRTED---
                Consensus  PS. .dprv. ..!stFP.g  hRsfFSDs%p .l...   .. .......... .......elI SAVD          i.%...l i%SkV..tET .   sgWFlc SPfr!D$l#p  k#.vptp.. ....... ..
        Conserved regions
```

Figure S2. (continued)

```
                             261                                                                                                                                                    390
CFB [Arabidopsis thaliana]   WKSDLEENLS LSWILIDPTC KRA-ADVSTR KPVSVHRHWL TGEVHVKFSS IFVVGNKKR- S-EQVEFTVT VVLAVFNRRE EETAVMQIRE VSLVAEDKDG RNLGGKVSLE ILVAAMGMKR R------FRA
Arabidopsis lyrata           WKSDLEKDLS LSWILIDQTG KRA-ADVSTR KPVSVQRHWL TGEVHVKFST IFVVGNKKR- S-EQVEFTVT VVLAAFNRRE EETAVMQIRE VSLVAEDKDG KNLGGNGSLV ILAAAMGRKR R------FRA
Capsella rubella             WKSELEENLS LSWILIDPTR KRA-ADVSTR RPVSVERHWL TGEIHVRFSN IFLVSGKKKP S-EEVEFTVT VVMAAFSRKG EEKAEVQIRE VSLVAEDAEG KNLGGKGSLV ILASAMGMDR RR-----FRV
Camelina sativa              WKSDLEENLS LSWILIDPIR KRA-ADISTR RPVSVERHWL TGELHVKFST VFVGGDKKKS Q-EEVEFTVT VVLAAFNRRG EEKAEVQIRE VSLVAEDMEG KNLGGKGSLV ILSSAMGMKR RR-----FRA
Tarenaya hassleriana         WVRNAEENLS LSWILIDPTR KRA-ANVSSR RPVSVHRHWL TGEVHVRFST VLAGNRKRKS S-SSSEL--- VEFATEMVMA PEASAVEVRE VSLVAEDMEG KNLGGEGSLV ILTAAMGGGR R-------RG
Arabis alpina                WKDDLEENLS LSWILIDPTC KRA-ADVSTR KPVSVQRHWL TGEVHVRFST ILVVG----- E-EQVEFTVT VVLAAFNRRE EDTATMQIRE RSVGGGG
AT2G27310                    WVKDMEESMK LNWILIDPIK KRA-ANISSR KAVSARRNWL TGDLEIRFST VVTAEAAEVA A--VVSCGSA EAWKEVDEEV GGEI--HVRD VRLQVEDIEG KCMKGRDSLV ILQGLLDGKR SC----KDDE
Ziziphus jujuba              WLKHLEENLT LSWIVIDPTR KRA-ANLSSR KAVSVQRHWL TGDVQLRYTT IMARGPGRD- D-DHVECAMV VTC--GGKE- GGKL--EVRE VSLQVEDMEG KHLSGRDSLG ILESAIEGGK RK----KLAG
Morus notabilis              WLKHLEENLK LSWIVIDPTR KRA-ANLSGR GAVSVVRHWL TGEIQLRYAT IMDGPASTPA T-EHVQCGMM VTC--GGEE- GDEV--RVRE VGLHVEDTEG RHLSGQESLV VFRNAIEGGK RK----KENG
Eucalyptus grandis           WLRHLEESLS LSWIVVDPAR KRA-ANFSTR RPVSVQRHWL TGDVQVQYVT VVAGGPPGSA A-EHVQCTAT VTC--GGKE- GGGL--HVRE VSFQVEDMEG KSLSGKDSLA ILGEAMESGE RK----KAKR
Vitis vinifera               WLSHLEDTLT LSWILIDPTR KRA-ANLSSL RPVSVKRHWL TGEVQVRYAT VV-GGDRTVG S-EVVQCGIV VTC--AGKE- GGEM--QVRE VNLQVEDMEG RHLNGKESLV ILQEALENGR RK----KAKG
Gossypium hirsutum           WLQHLEENLS LSWIVINPTR KKA-VNVSSR RAVSVQRHWL TGDVQVRFGT VTAGDEGRGS SRELVECGVV VTC--CGKE- GGEM--HVRE VCMVMEDMEG KGLNGKDSLV ILEGVIEQGR RK----GGEG
Theobroma cacao              WLKHLEENLT LSWIVIDPSR KKAAVNMSSR RAVSVKRHWL TGDVQVRFGM VMGGGGRRGS SRELVECGVV VTC--VGKE- GGEM--HVRE VSMVMEDMEG KGLNGKDSLV ILEGVMENGR RK----KGNG
Fragaria vesca               SLKHLEENLT LSWIVIDPAQ KRA-ANLSSR KPVSVTRHWL TGEVQLRFAM IMPGNRRG-- --EFVQCGMV LTC--KGSD- GGKL--HVRE VSMQMEAMEG NHLNGKESLV ILQGAIEGGK RK----R-ES
Malus domestica              SLKHLEDNLS LSWIVIDPTR KRA-ANLSSR TAVTVQRHWL TGEIQLRFAT ILAGEKKG-- --EYVQCGMV VTCKRSGSE- GREL--HVRE VSMQMEGMEG NHLNGRESLV ILQRAIEGGK RR----K-EV
Prunus persica               SLKQLEENLS LSWIVIDPTR KRA-ANFSSR RAVTVQRHWL TGEIQLRFAT ILAGEKRG-- --EFVQCGMV VTC--NWSE- GGEL--HVRE VSMQVEGMEG NHLNGKESLV ILERAIEGGE RR----K-EV
Ricinus communis             WLKQLEEDVT LSWILIDPEQ KRA-VNISSK RPVSVQRHWL TGEVQAKFAT IFAGDRGKGS ETEYVECEVV VTC--GGKE- GGEV--RVRD VSMGMEDMEG KALTGEDSLV ILKEATERGE RR----RGE-
Jatropha curcas              RLKQVEQNLT LSWILIDPKQ KRA-VNISSH RAVSVQRHWL TGEVQAKFAT ILAGDEEKGS EREYVQFEVV VTC--GGKE- GGEI--HVRD VSMVMEDMEG KTLTGKESLV ILQEAMERGE RR----KGKS
Manihot esculenta            WLRQLQENIT LSWILIDPQK KRA-MNMSSQ RAVSVRRHWL TGEVQVKFAS ILAGDGGMGS ERESVQCEIM VTC--GGEE- GGEV--HVRD VSMVMEDMEG KVLSGKESLV IVDGAMERGE RR----KEKS
Populus trichocarpa          FVKQLEENMT LSWILIDPKR RRA-MNLSSG RPVSVQRHWL TGEVVVKFAT IMAGD---GG EKEFVECGVM VCC--GKKE- GGEM--EVRE ISMGMEDMEG RNLTGKESLV VIQEAMERGE RR----KGKC
Nelumbo nucifera             TCQDLAENLK LSWIVIDATG RRA-ANLSSW RPVSVQRHWL SREIHARFAT ILAG-DR-GS T-EFVQCGIV VTC--GGCE- GGEM--QMRE VSLQVEDMDG TNLNGKDSLV IMQRAMQRGE RK-----KGR
Citrus clementina            TCRELAEDLT LSWILIDPIG RRA-MNLSSY KPVTVQRHWL SGEAHVRFSL VLSGGDK-GS SSEFVQCGIV VTC--GGSQ- GGEM--HVRE VNLQVEDMDG TNLNGKDSLV ILQRGLEGKR RN----VKGR
Cucumis melo                 WLGHLEENLT VSWIIIDPIN NRA-ANISSR QPVKVRRHWL SGEIQVQYTT VMGGDRRAGS AVEMVECAVV VSCGEKEEE- GMEM--SVTE VSMQMLDMEG KHLNGKESLG ILREAMEKGK RI----RGRK
Daucus carota                FRSQLDQNLT LSWILVDPVS QRA-ANLSTL TPVSVERHWL DEDIHAHYVT SLPG-LFAGE SSEVVQCSIL VVC---GGT- GGDL--QIKE VSLLVQDMDG KSLNGRESVA ILHEAMEGQR KR----KGKR
Dorcoceras hygrometricum     CMQIASDRLR VSWILIDPAK SRA-VSVASL KAVEARRHWL TEEVLLRFAT VASGGDD--- --ELFQCAVM LTF--GGRE- GKQM--HARE VYLQVEDVEG KIVRGMESLG ILREAMEGPR CI----SSQR
Arachis duranensis           WLRHVQENLE LSWILIDPSL KRA-ANLSSR RAVSARRHWL TGEAEVVYAV GVEEGG---- ----AQCAVK VTC---CGKS GGEM--QVRE VSLTMEDLDG RHVMGRDSMV ILQRAMESSK RK----KVDV
Vigna radiata                WLAHLEENLG LSWIVIDPTR KRA-ANLSSR RPVAARRHWL TGDLEVIFAV TMET------ ----VQWVIR VTC---CGKA GGAM--HVRE ASLTMEDTEG RHVTGRDSFV ILQDAVENGE RR----KPDP
Phaseolus vulgaris           RLAHLEQNLE LSWIVIDPTG KRA-ANLSSR RPVAARRHWL TGDLEVSFAV TMES------ ----VQCVIR VTC---FGKA GGAM--HVRE VGLMMEDTEG RHVIGRDSFV ILQDAMANGE RR----KPDP
Glycine max                  LLNHLVENLA LSWIIIDPTR KRA-ANLSSR RPVSARRHWL TAELEVLYAV PMET------ ----VQCVVK VTC---CGKV GGAM--HVRE VSLTMEDTEG RHVMGRDGVV ILQDAMENGK RE----KLDA
Cynara cardunculus           MQSNLEKHMT LSWILIDPIQ HRA-VNLSSI KPVSVHRNWL TDDIELTFAV VTAT------ ---YVKCNIE MTC--GVKEG SGEV--YVSG VSLTVQDVDG KCLNGKDSMV ILQGLAVAQR RS----RKHS
Sesamum indicum              --SDMVDDMM LSWILIDPIG RR-AVNLSSH KPVSVERHWL TGEVQVRYAS ILAVDQR--- ---QVQCGIV IRC--GGSEC GDEM--QVRE VSLEIEDMDG THLNGKESLV FLQGALEGKK GT------GK
Erythranthe guttata          ACAEMIDDMV LSWILIDPTG RRRAVNLSSH KPVAVQRHWL SGEVQVRYAS ILAADQG--- ---HVQCGIV VTC--AASE- GGEM--QVGE VSVEMEDMDG NHLTGKDSLV FLHGALEGKK GT------GK
Beta vulgaris                TCKDLYADLE LSWIVMDPTG GR-AVNVSSF RPVSVERHWL SGEVHVRYAA VV---EG--- ---HVMCSVE VTC--VGGGP GGGLVLHVTE ACLKMEDVDG AHLNGKDSLV ILQRVLEGKR GSLCRKGGME
Medicago truncatula          TCHDLGEELR LSWILIDPAS HRA-VNVSSR KAVAVQRHWL SGEVKARFTT VVYG-ET-AT ALEVALCSVV VML------- -AEESMEVRE VSLQVEDMDG KHVNGRDSLV ILQKALEGGR KW---KGVGE
Cicer arietinum              TCRNLGEELR LSWILIDPVS RRA-VNVSSG KAVTVQRHWL SGEVNLQFAT VVYG-EI-AT ALEVALCSVV VTL------- -VEESMEVRE VSLQVEDMDG KHVNGRDSLV ILKRALEGGR NW---KGDGE
Cajanus cajan                ECAEIGEKLR LSWIVVDPEG RRA-VEVSGG RAVSVERHWL SGEVRVRLAT VVGGER-GS ATEAALCSVT VTF------- -GGE-MQVRE ACLEMEDLDG MQLNGKESLG ILQRALEGKR GKLLSEGHHG
Nicotiana tabacum            CKSRLKENMK LSWILIDPKK NRA-VNMSSL KPVSVRRHWL TGELKVRYST VMASGADAGD GGGLVQCGIV VTC---EGKE GGEL--HVRE VSMQMEDMDG KVLCGKDSLV ILQEAMEGGR KKRKEGEEKE
AT2G36090                    TCRDLEKDLT LSWIVIDPIG KRA-ANISSH RPVSVQRNWI SGEVEAQFAT VVGA------ ----VECVIT VVT------- CGEEEMHVRE VSLKVEKMEG THLNGRDSLV ILRSVMEGKR VN------GS
Consensus                    ....leen$. LSWIl!DP.. kRA.a#.Ssr rpVsv.RhWL tg#v.vr%at ...g...... ..e.v.c... vtc....... gge....!r# vs$..#dm#G ..l.GkdSlv !l..a.eggr r.     .....
Conserved regions
```

Figure S2. (continued)

```
                             391                                                                         461
CFB [Arabidopsis thaliana]   GGEEEGKEKY IEYMERKTAK AEMKWRRGKE TAMETAACWI A---VLLLGF LLCFYLFMHK NLVAIMKKLI K
        Arabidopsis lyrata   GGEGGGKEKY LEFMERKSEK AEMKWRRGKE TAMETAACWI A---VLLLGF LLCFYLFIHK NMVAIMKKLI K
          Capsella rubella   GREEEGKEKY REFKERKTAK SEMKWRPEKE AAMETAACWI A---VFFLGF LLCFYLFIHK NMMAIMNKLM K
           Camelina sativa   GGEEDGKEKY REFMERKTGK AV---RREKE TAIETAACWI A---VLLLGF LLCFYLFIHK NMIAIMNKLM K
      Tarenaya hassleriana   GGE----ERY RELEQRKREA REEKGRRRSS KAVDTAACVV AGGAAVVVSF WLCLYLCIRQ KLVVIVNKLK
            Arabis alpina
                 AT2G27310   ERR--AKERY EEYVRMKIQW RENKERREKA QDTICMIFGF S---MFVLLW SFILLR
            Ziziphus jujuba  KGREFGKQRY EEYLEMQRQR KEKKARRERA LDMACIVCGI S---MFVGFW TFLVFR
           Morus notabilis   VGE--GKERF EEFLVKRREM REKKQRRERA LDLACILTGV S---IFVAFW SFVLFR
        Eucalyptus grandis   ASE--GKERF EEYEARKRER RERKQRAERA LDLLCIVVGV T---AFVGFW SFILFR
            Vitis vinifera   EE---GKARY ERYLEMMGER RERRLRREKT LDMVCIALGV T---IFLSFW SYFLFT
        Gossypium hirsutum   NE---GKVKF EEFQERKRGR KEENQRKERV LDLVCITVGV V---GFVSFW SAILFK
           Theobroma cacao   NE---GKERY EEFGERKRER KERRQRKERA LDLVCISIGV A---GFVTFW SAMLFR
           Fragaria vesca    EEGGEGKARF GEFLEMKRER KERTQRRERA LDMVCIAAGV T---LFMAFW SFVLFR
           Malus domestica   K----GKAKY EEYLEMKRER RERKERREKA LDMICIAAGV T---IFMAFW SFVLFR
           Prunus persica    K----GKGRF EEYLEMKRER RERKERREKT LDMICIATG
          Ricinus communis   --D--GKERY KEFLKRKREW KERKERRESV LDWVCIATGV T---SFLAFW SFILFR
          Jatropha curcas    GKE--GKERF KEFLERRKER KERIQRREKI LDWVCIASGI S---SFMAFW SYILFQ
         Manihot esculenta   GEE--GKERY NEFVGRRRDR RERKHRREKI WDLVCIASGF S---SLMAFC SFLLLS
       Populus trichocarpa   GTE--GKGRY EEFVERKRER KARKQKMEKV LDMVCIVTGI T---IFVSSW SFILFR
          Nelumbo nucifera   KGE--GKERY EELLRRKAER KERKLRREGR LDSMCMAFSV L---FFASVW MLCLFR
        Citrus clementina    EDE--GKRKY EDYVEKKRER REKKLRTEGA LDMLCVAFGV F---TFFSSG LYLLWR
              Cucumis melo   GD---GKLRY EEYEEMKRKR KARKERIENG LDMLCRSSG
             Daucus carota   EEE---RERY QELMEMRREM GERKQRRERM MDMVCMLTGV S---IFMAFC GFLWLR
Dorcoceras hygrometricum     ME----KEKY EMLVKMKIEC LERKQRRERS LDMAFVAIAV S---IFLAIL IYFLNR
        Arachis duranensis   VE---AKERY EKFCDTKIER REMRIKREKT MDTVAMFVAF T---VFVTLF CFLRFYVCV
            Vigna radiata    EE---AKARF DKFCRLKREI RERKLRRDSA MDTVAMLVSF T---IFVSLF WFMVFGF
        Phaseolus vulgaris   EE---ARARF EKFCRLKKDI RERKVRRDNA MDMVAMLVSF T---VFVSLF WFMVFGF
               Glycine max   EK---AKERF EKFSIVKREI RERKMRRDRA MDMVAMLIAF A---VFALLF CFMAFGV
        Cynara cardunculus   GGGEEERDRY KEYIQRRRER DEKTERRERR LDMACVVSGV A---FFVAFW SFALF
           Sesamum indicum   NRVEEGQRRY KAYVEMKRER KERKLRTEGA LDMVCVAFGV S---IFVAFC ---CFLFSR
       Erythranthe guttata   NRVDEGQRRY CKYMEMKRER RERKLRVESA LDMFSVAFGI S---LFVGFC ---CFLLCR
             Beta vulgaris   GRVEEGRRRY LEYLERKRER REKVLRREGR LDNMCVGIGV F---LFVGII YLLCFSTSR
       Medicago truncatula   G------EGY REFVKKKKER EERKKRVERR MDIMCLFVGL V---SLTFVA FFAL
           Cicer arietinum   -------EGH REFVKKKKER EERKKRAERR LDMLCLCVGL V---SLTFVA FFAL
             Cajanus cajan   N------DRF ADFVYRKMER KERKVRDERR LDLLCLCLGF A---ILSFAA LSTLFL
         Nicotiana tabacum
                 AT2G36090   RREVESKKRH EEFMEKKREM KEKKMRVESV FDILTVAFGI L---GFVLLV VFCLWRTSI
                 Consensus   .....gk.ry .e....krer .erk.rre.. ld..c...g. .   .f.... ...lf..... ..........
         Conserved regions
```

Figure S2. (continued)

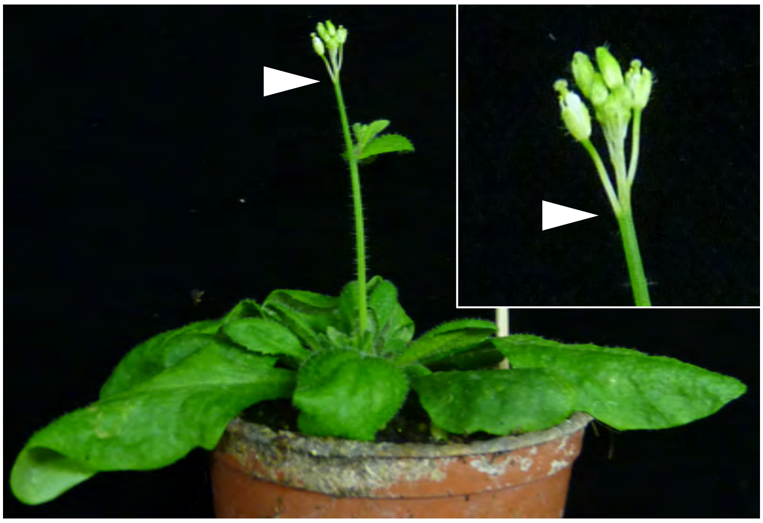

***Pro35S:CFB-GFP*-52V**

Figure S3. Phenotype of plants overexpressing a *CFB-GFP* fusion gene. Photograph of a transgenic plant harboring the *Pro35S:GFP-CFB* gene (individual 52V, see Figure 3) four weeks after germination showing the typical CFB overexpression phenotype with white inflorescence stem tips. The arrowhead indicates the onset of the white stem tissue. The inset shows the inflorescence of the same plant at a higher magnification.

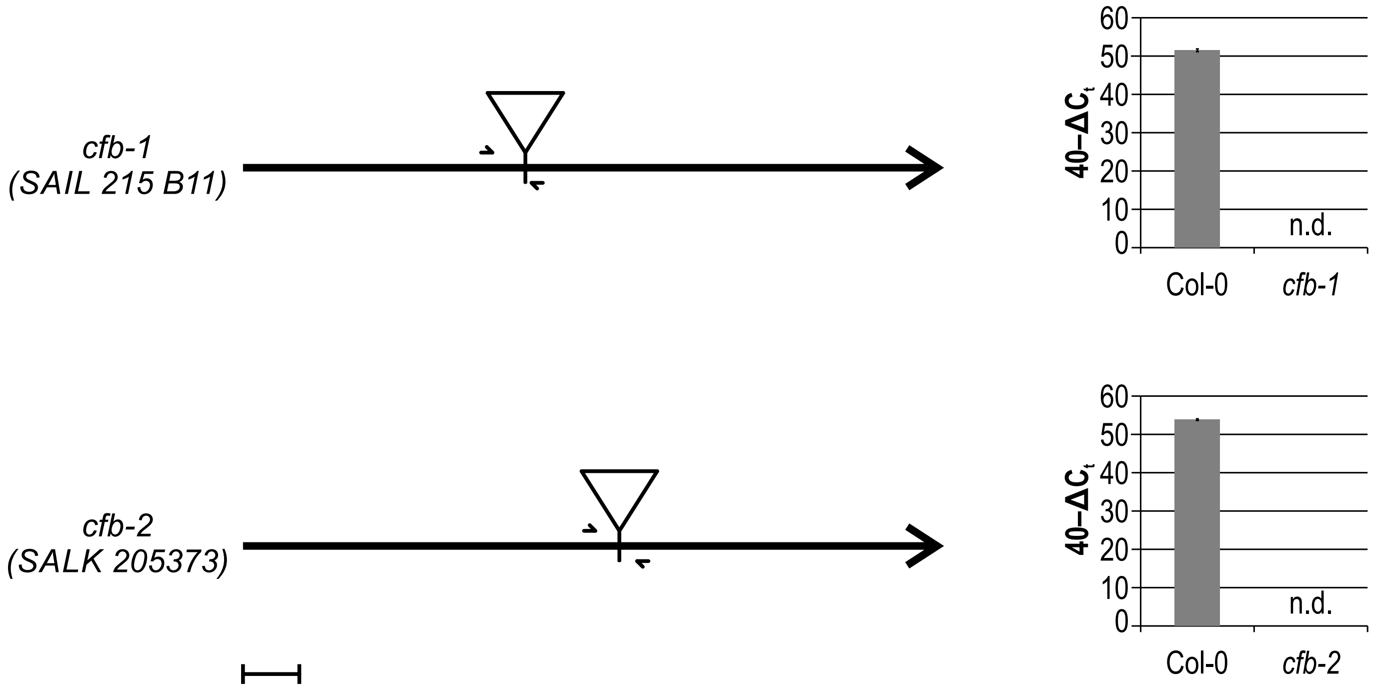

Figure S4. Analysis of the *CFB* transcript in *cfb-1* and *cfb-2* mutants. The position of the T-DNA insertions relative to the cDNA of *CFB* (represented by a bold arrow) is marked by triangles, the position of the primers used for quantitative real-time RT-PCR by half arrows. Transcript abundance was measured by 40 cycles of quantitative real-time RT-PCR in leaves of wild-type (Col-0), *cfb-1*, and *cfb-2* mutant plants. *AT3G25800* (*PP2AA2*) was used as a reference gene. Primer sequences are listed in Table S2. Error bars = SD (n = 3). n.d.: not detected. Scale bar = 100 bp.

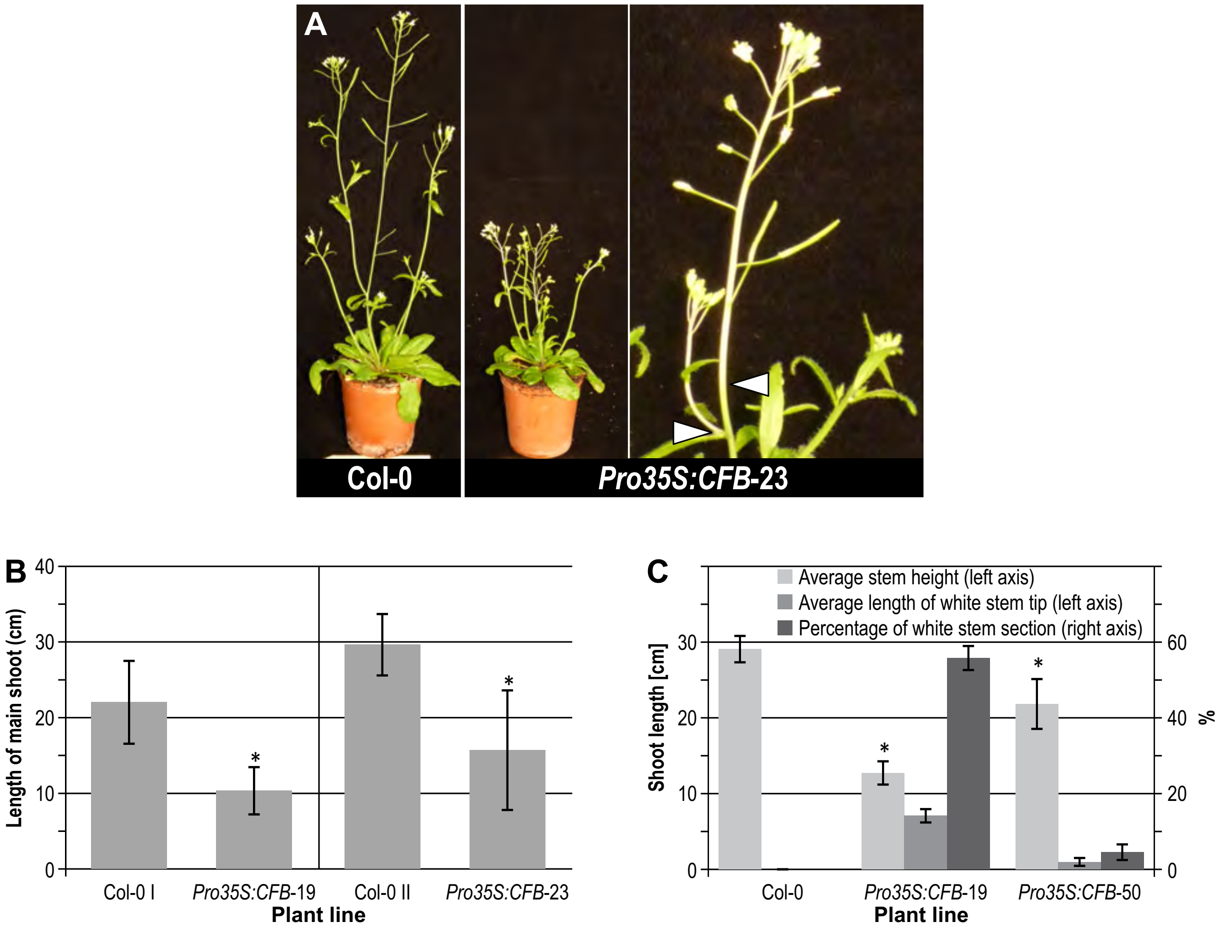

Figure S5. Comparison of independent *CFB* overexpressing lines to the reference line *Pro35S:CFB*-19 and wild type. A, Comparison of line *Pro35S:CFB*-23 to wild type at the flowering stage. The onset of albinotic tissue formation is indicated with arrowheads. B, Comparison of inflorescence stem length between the wild type (Col-0) and the *CFB* overexpressing lines 19 and 23, respectively. Stem lengths of plants grown on soil in the greenhouse were measured 30 (line 19) or 38 (line 23) days after sowing. C, Lengths of the main stems and percentage of the white upper portion of the stems of lines 19 and 50 in comparison to the wild type. Stem lengths of plants grown on soil in the greenhouse were measured 38 days after sowing. Error bars = SD (n ≥ 8). Asterisks indicate a p value < 0.01 (Student's t-test; comparison transgenic lines vs. wild type).

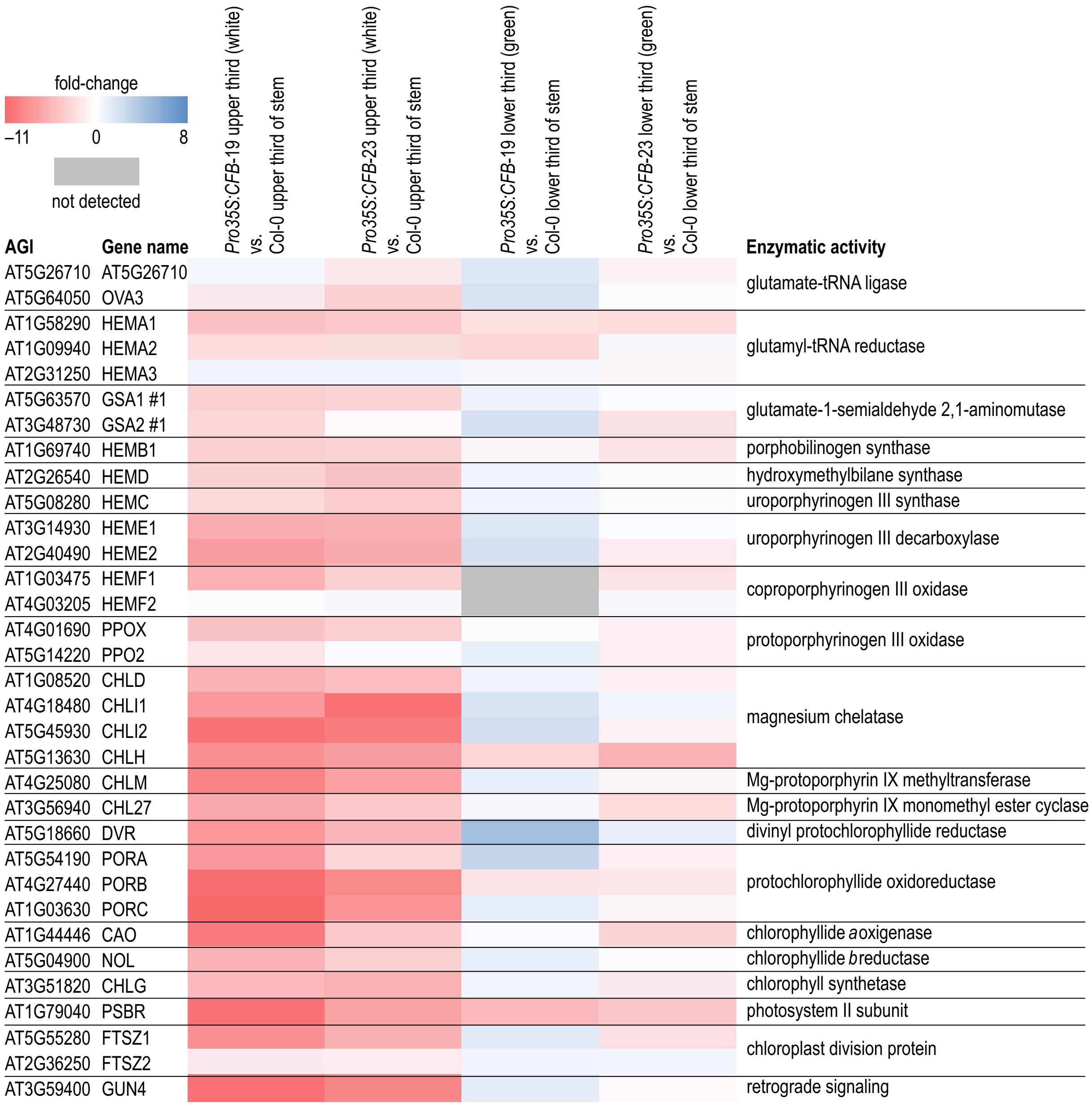

Figure S6. Expression of chlorophyll biosynthesis and other chloroplast-related genes in green and white stem sections of two *CFB* overexpressing lines, *ProCFB35S:CFB*-19 and *Pro35S:CFB*-23. The upper and lower third of the stems of flowering plants were pooled from twenty plants of wild type and the two *CFB* overexpressing lines each. Transcript levels were quantified by qRT-PCR. The fold-changes between the samples indicated were calculated and shown as a heat map.

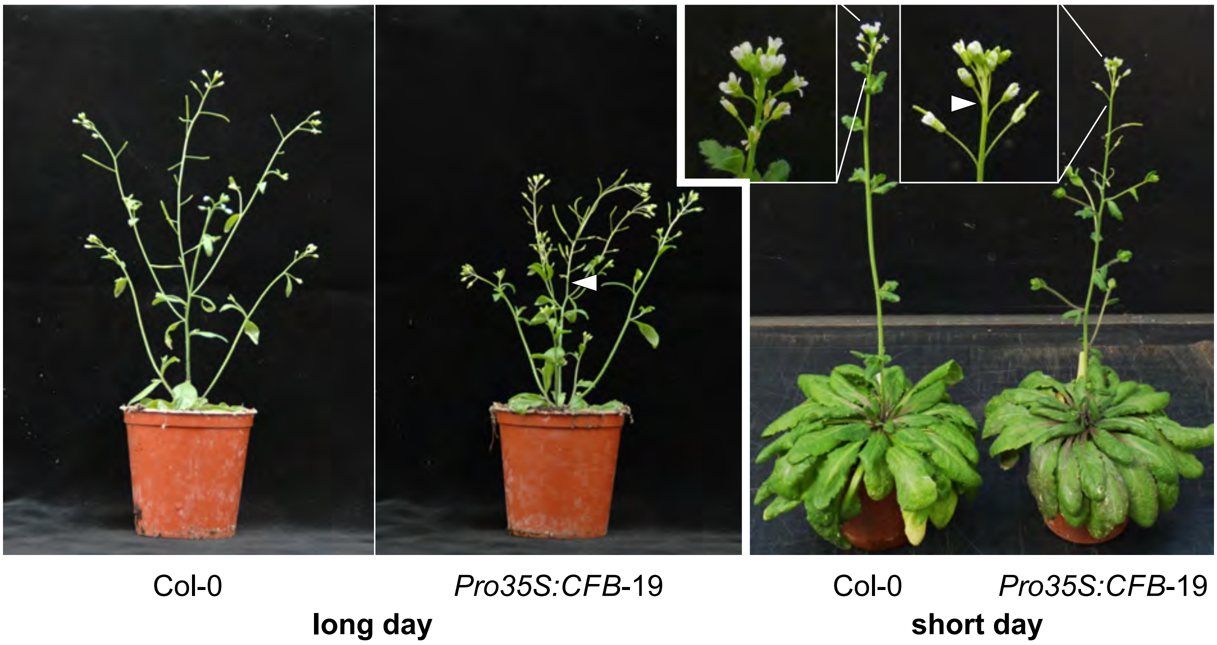

Figure S7. Formation of the albinotic stem tip of *CFB* overexpressing plants grown under long-day (16 h light/8 h dark) and short-day (8 h light/16 h dark) conditions. Arrowheads mark the onset of albinotic tissue formation.

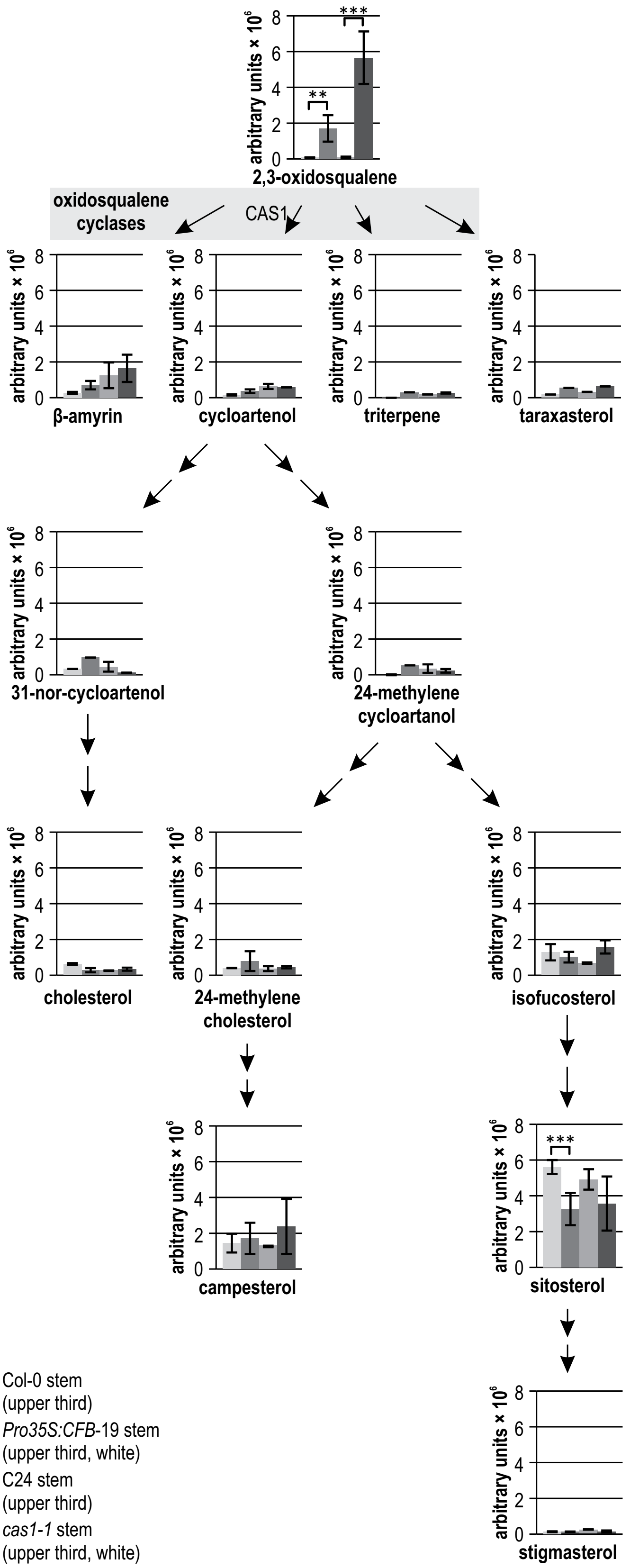

Figure S8. Relative concentrations of sterol metabolites in different genotypes and tissues. Sterol metabolites were measured in the upper thirds of the inflorescence stems of wild-type, *CFB* overexpressing (Col-0 background), and *cas1-1* (C24 background) mutant plants. In the two latter plants, the upper third of the stem contained only albinotic tissue. Units of the y axis are mass spectrometry peak heights normalized to the internal control × 10^6. Significance codes: **: $p < 0.01$; ***: $p < 0.001$. Error bars = SD of two to four biological replicates
