# Supplementary material for: Analysis of CFB, a cytokinin-responsive gene of Arabidopsis thaliana encoding a novel F-box protein regulating sterol biosynthesis
Source: J Exp Bot. 2017 May 12;68(11):2769–85. doi: 10.1093/jxb/erx146 (PMC5853388; doi:10.1093/jxb/erx146)
Supplement: supplementary_tables_S1_S2 [file erx146_suppl_supplementary_tables_s1_s2.pdf]

Supplemental Table 1. Cloning procedures and PCR primers used in this study

No	Plasmid	Purpose	Cloning procedure	forward primer	reverse primer	Backbone reference
1	pDONR201–CFB-CDS	Intermediate for cloning of plasmid 2	PCR product from cDNA of untreated Col-0 leaves cloned by BP into pDONR201	GGGGACAAGTTTGTACAAAAAAGCAGGCTTATCTTTTCTTGCATTTTCAACTCA	GGGGACCACTTTGTACAAGAAAGCTGGGTACCGATTCAATTCCTCTTTGAA	Invitrogen, Carlsbad (CA), USA
2	pB2GW7–CFB-CDS	Plant transformation vector for overexpressing CFB; used for transformation of *A. thaliana*	Insert of plasmid 1 cloned by LR into pB2GW7			Karimi et al., 2002
3	pDONR221–CFB	Intermediate for cloning of plasmids 11, 19 and 25	PCR product from cDNA of untreated Col-0 leaves cloned by BP into pDONR221	AAAAAGCAGGCTATATGCTTTCGTCTTCGTCTTC[1]	AGAAAGCTGGGTGCAATTCCATTGAAGTTTTGAATC[1]	Invitrogen, Carlsbad (CA), USA
4	pDONR221-CFB ΔF-box	Intermediate for cloning of plasmids 12 and 20; contains the coding sequence of CFB lacking the N-terminal 74 amino acids including the F-box domain	PCR product from cDNA of untreated Col-0 leaves cloned by BP into pDONR221	AAAAAGCAGGCTATATGGATACACGTGTCCAAAGC[1]	AGAAAGCTGGGTGCAATTCCATTGAAGTTTTGAATC[1]	Invitrogen, Carlsbad (CA), USA
5	pDONR221-CFB ΔTM	Intermediate for cloning of plasmid 13; contains the coding sequence of CFB lacking the C-terminal 38 amino acids including the annotated transmembrane domain	PCR product from cDNA of untreated Col-0 leaves cloned by BP into pDONR221	AAAAAGCAGGCTATATGCTTTCGTCTTCGTCTTC[1]	AGAAAGCTGGGTCTACGTTTCTTTCCCTCGCC[1]	Invitrogen, Carlsbad (CA), USA
6	pDONR221-without stop CFB	Intermediate for cloning of plasmids 17, 18, and 22; contains the coding sequence of CFB lacking stop codon	PCR product from cDNA of untreated Col-0 leaves cloned by BP into pDONR221	AAAAAGCAGGCTATATGCTTTCGTCTTCGTCTTC[1]	AGAAAGCTGGGTATTTTATTAGTTTTTTCA[1]	Invitrogen, Carlsbad (CA), USA
7	pDONR221-without stop CFB ΔF-box	Intermediate for cloning of plasmid 23; contains the coding sequence of CFB lacking the N-terminal 74 amino acids including the F-box domain and missing a stop codon	PCR product from cDNA of untreated Col-0 leaves cloned by BP into pDONR221	AAAAAGCAGGCTATATGGATACACGTGTCCAAAGC[1]	AGAAAGCTGGGTATTTTATTAGTTTTTTCA[1]	Invitrogen, Carlsbad (CA), USA
8	pDONR221-without stop CFB ΔTM	Intermediate for cloning of plasmid 24; contains the coding sequence of CFB lacking the C-terminal 38 amino acids including the annotated transmembrane domain and missing a stop codon	PCR product from cDNA of untreated Col-0 leaves cloned by BP into pDONR221	AAAAAGCAGGCTATATGCTTTCGTCTTCGTCTTC[1]	AGAAAGCTGGGTACGTTTCTTTCCCTC[1]	Invitrogen, Carlsbad (CA), USA
9	pDONR221-pCFB	Intermediate for cloning of plasmid 25; contains the 2000 bp genomic region upstream of the CFB transcription start site	PCR product from genomic DNA of Col-0 leaves cloned by BP into pDONR221	AAAAAGCAGGCTGTCCTGTCAATATAAGAG[1]	AGAAAGCTGGGTGAGATAAAGATTGAGTTG[1]	Invitrogen, Carlsbad (CA), USA
10	pDONR221-ASK1	Intermediate for cloning of plasmids 14 and 15.	PCR product from cDNA of untreated Col-0 leaves cloned by BP into pDONR221	AAAAGCAGGCTATATGTCTGCGAAGAAGATTG[1]	AGAAAGCTGGGTTCATTCAAAAGCCCATTGG[1]	Invitrogen, Carlsbad (CA), USA
11	pBTM-116cD9-GW-CFB		Insert of plasmid 3 cloned by LR into pBTM-116cD9-GW			Stelzl et al., 2005
12	pBTM-116cD9-GW-CFB ΔF-box		Insert of plasmid 4 cloned by LR into pBTM-116cD9-GW			Stelzl et al., 2005
13	pBTM-116cD9-GW-CFB ΔTM		Insert of plasmid 5 cloned by LR into pBTM-116cD9-GW			Stelzl et al., 2005
14	pACT2-ASK1		Insert of plasmid 10 cloned by LR into pACT2			Clontech, Mountain View (CA), USA
15	CD3-1737 (NX32_GW)–ASK1 [NubG]		Insert of plasmid 10 cloned by LR into pNX32_GW			Lalonde et al., 2010
16	CD3-1739 (NWT-X_GW) [NubI]					Lalonde et al., 2010
17	CD3-1740 (MetYC_GW)–CFB [Cub]		Insert of plasmid 6 cloned by LR into ppMetYC_GW			Lalonde et al., 2010
18	CD3-1740 (MetYC_GW)–CFB ΔF-box [Cub]		Insert of plasmid 6 cloned by LR into pMetYC_GW			Lalonde et al., 2010

No	Plasmid	Purpose	Cloning procedure	forward primer	reverse primer	Backbone reference
19	pk7WGF2-CFB	Used to express CFB fused C-terminally to GFP in transiently transformed *N. benthamiana* leaves	Insert of plasmid 3 cloned by LR into pk7WGF2			Karimi et al., 2002
20	pk7WGF2-CFB ΔF-box	Used to express the CFB deletion construct lacking the N-terminal 74 amino acids including the F-box fused C-terminally to GFP in transiently transformed *N. benthamiana* leaves	Insert of plasmid 4 cloned by LR into pk7WGF2			Karimi et al., 2002
21	pk7WGF2-CFB ΔTM	Used to express CFB deletion construct lacking the C-terminal 38 amino acids including the annotated transmembrane domain fused C-terminally to GFP in transiently transformed *N. benthamiana* leaves	Insert of plasmid 5 cloned by LR into pk7WGF2			Karimi et al., 2002
22	pk7FWG2-CFB	Used to express CFB fused N-terminally to GFP in transiently transformed *N. benthamiana* leaves	Insert of plasmid 6 cloned by LR into pk7FWG2			Karimi et al., 2002
23	pk7FWG2-CFB ΔF-box	Used to express the CFB deletion construct lacking the N-terminal 74 amino acids including the F-box fused N-terminally to GFP in transiently transformed *N. benthamiana* leaves	Insert of plasmid 7 cloned by LR into pk7FWG2			Karimi et al., 2002
24	pk7FWG2-CFB ΔTM	Used to express CFB deletion construct lacking the C-terminal 38 amino acids including the annotated transmembrane domain fused N-terminally to GFP in transiently transformed *N. benthamiana* leaves	Insert of plasmid 8 cloned by LR into pk7FWG2			Karimi et al., 2002
25	pKGWFS7-pCFB	Used to express a GUS-GFP fusion protein driven by the 2000 bp genomic region upstream of the transcription start site of CFB as a promoter	Insert of plasmid 9 cloned by LR into pKGWFS7			Karimi et al., 2002

1) PCR products obtained using these primers were completed in a second round of PCR using the primers GGGGACAAGTTTGTACAAAAAAGCAGGCT (forward) and GGGGACCACTTTGTACAAGAAAGATGGGT (reverse) before cloning them by BP into the respective pDONR vectors.

References

Karimi M, Inzé D, Depicker A (2002) GATEWAY™ vectors for Agrobacterium-mediated plant transformation. Trends in Plant Science **7:** 193-195

Lalonde S, Sero A, Pratelli R, Pilot G, Chen J, Sardi MI, Parsa SA, Kim D-Y, Acharya BR, Stein EV, Hu H-C, Villiers F, Takeda K, Yang Y, Han YS, Schwacke R, Chiang W, Kato N, Loqué D, Assmann SM, Kwak JM, Schroeder J, Rhee SY, Frommer WB (2010) A membrane protein / signaling protein interaction network for Arabidopsis version AMPv2. Frontiers in Physiology **1:** 24

Stelzl U, Worm U, Lalowski M, Haenig C, Brembeck FH, Goehler H, Stroedicke M, Zenkner M, Schoenherr A, Koeppen S, Timm J, Mintzlaff S, Abraham C, Bock N, Kietzmann S, Goedde A, Toksöz E, Droege A, Krobitsch S, Korn B, Birchmeier W, Lehrach H, Wanker EE (2005) A Human Protein-Protein Interaction Network: A Resource for Annotating the Proteome. Cell **122:** 957-968

Supplemental Table 2. qRT-PCR and sequencing primers

AGI	Gene name	forward	reverse
AT5G26710	AT5G26710	CGTCTCTCTCTCAAACCCAC	CAGCGGCGGAAGAATCAA
AT5G64050	OVA3	AGAATCCGAGGGGAGTTTTG	AGTTTGTCTTCCACAGCAGG
AT1G58290	HEMA1	AGCTCGAGAAGTGCATGTCC	CGCTCAGCGTTCTACTGTCA
AT1G09940	HEMA2	CCTGTTGAGATGCGTGAGAA	CGATGCTGAGATAGAGCCAAA
AT2G31250	HEMA3	GCAGCATTGTGGTGATTGG	GGTAACGAGAAAGAGCGGA
AT5G63750	GSA1 #1	TCAATCATAAGCCCACCCAC	GAGGAACACGAAAACCCCAA
AT3G48730	GSA2 #1	GGGCTCACGAATAAGAGACA	GCCAAAGCAGCAAGAACC
AT1G69740	HEMB1	ATGGATGGGCGAGTAGGT	CTCTCTGTAGTTTGCTGGGT
AT2G26540	HEMD	GCACTATCTGCTCCTGTTCT	TCAAGTCCTGGTTTCTCTGG
AT5G08280	HEMC	CAAGGACGAAGAAGGCAAC	GACCAGCACGAGATAGCA
AT3G14930	HEME1	GAGCGAATGAAAGGAACTGG	AGGAAGCGGAGAGAATAGGT
AT2G40490	HEME2	AAACTCACCCAAACCTACCTC	TTCCTTGAACTGCTATGTCTCT
AT1G03475	HEMF1	ATTCTCGTCTCTCTTCCGCT	CCACTCCTTCGGGTTGATG
AT4G03205	HEMF2	GCAAGACGGAAATGTATGGG	GCAAAAGGGTTCTGAGGATG
AT4G01690	PPOX	CCGAAAGAAGCAATCCGAAC	GTGTTTGTAGACCCGCCA
AT5G14220	PPO2	GCTGATGGAAGAGTAGGTGG	GTTTCTCACGAAGCCCAAGA
AT1G08520	CHLD	TCGTGTGGCGAAGTGTTTAG	CGCTATTTTGTGGAGGAGGT
AT4G18480	CHLI1	TTCTGGTTGGAATACGGTTGA	CTTGTCCTGCTCGGTTTTGT
AT5G45930	CHLI2	TGTGATTGACCCGAAGATAGG	TCAGTAGCACCCAAAGGAAGA
AT5G13630	CHLH	CCTCAATGTGTTTGCTTCCA	GTTTCCCCCAGTTTTCTTCC
AT4G25080	CHLM	CCGTTTGCTCCTTCCTTGTTG	GAGTGCTGCTACGGATCCTC
AT3G56940	CHL27	CAGAATGTCAGCCTCTTCCT	AAGTCCGTCGTGTAGAACC
AT5G18660	DVR	CGCCGCAGAGAGTATGTT	TTCACCGAGTTCTTGACCC
AT5G54190	PORA	TTGGTCTCCTCTGCTTTCTCTGTCC	TGCTCCGAAAGTGAAACACCGAAC
AT4G27440	PORB	GTGCCTCCATTACCGACCAA	TGCCGTCCACGGATTTTGTA
AT1G03630	PORC	GGCTCTCCAAGCTGCCTATT	CTTGATGGTCAACAGGGTGGA
AT1G44446	CAO	GATAAACCCTCCTCTTCAACCA	TCCCGTCTTCACCCCTAAA
AT5G04900	NOL	TGGAATCAAAGCCTACACCA	TGAACAAACTGAGAAAGCAATACA
AT3G51820	CHLG	GTCTCCACCATCCACTCTTC	GCACGCACAACAAATCTCC
AT1G79040	PSBR	AGAGGATTACCGTCGCTCACAAGA	ACACCGTATCCCTTGCCCTTTCT
AT5G55280	FTSZ1	CAATGCTCGGGGTAGGTGTT	TGCCAAACTTGTCACGACCT
AT2G36250	FTSZ2	CCTCAGCGGTCAAGTAAGCA	TGACGCAGCATCTGCTTGTA
AT3G59400	GUN4	GCCAATCTCACTTCGGACCA	GTTGAAACGGCAGATACGGC
AT3G44326	CFB	ACACAAAAGGAAACCAAGTAAGAGG	GCCGTGTCCTCCGATGTTTT
AT5G53300	UBC10	CCATGGGCTAAATGGAAA	TTCATTTGGTCCTGTCTTCAG
AT3G25800	PP2AA2	CCATTAGATCTTGTCTCTCTGCT	GACAAAACCCGTACCGAG
primer pair spanning *cfb-1* insertion site		GTCCGTTGATACCTCGGAGT	GCAAAGGAACCAACCAGAGA
primer pair spanning *cfb-2* insertion site		AGGATGACACGTGGAAAAGTGA	TGCACCGAGACAGGTTTACG
LBa1 (sequencing of SALK T-DNA insertion lines)		TGGTTCACGTAGTGGGCCATCG	
LB3 (sequencing of SAIL T-DNA insertion lines)		TAGCATCTGAATTTCATAACCAATCTCGATACAC	
